# Supplementary material for: Associations of resuscitation fluid load, colloid-to-crystalloid infusion ratio and clinical outcomes in children with dengue shock syndrome
Source: PLoS Negl Trop Dis. 2025 Jan 10;19(1):e0012786. doi: 10.1371/journal.pntd.0012786 (PMC11756768; doi:10.1371/journal.pntd.0012786)
Supplement: S1 File — (DOCX) [file pntd.0012786.s002.docx]

**Indications for mechanical ventilation (MV) support in children with severe dengue shock syndrome (DSS)**

Indications for mechanical ventilation in children with severe DSS were based on the Vietnamese Ministry of Health Dengue Guidelines [1,2]. First, patients with severe DSS, in whom the initial management of nasal continuous positive airway pressure (NCPAP) fails, should be indicated for MV. Further indications for MV included severe DSS patients presenting with huge pleural and abdominal cavity effusions, abdominal compartment syndrome, pulmonary edema and/or fluid overload, acute respiratory distress syndrome (ARDS), and high volume of continuous intravenous infusion (≥ 7 ml/kg per hour) in many consecutive hours of infusion.

**References**

1. Guidelines for Diagnosis and Treatment of Dengue Infection (2019), the Vietnamese Ministry of Health. Available from: <https://thuvienphapluat.vn/van-ban/The-thao-Y-te/Quyet-dinh-3705-QD-BYT-2019-Huong-dan-chan-doan-dieu-tri-sot-xuat-huyet-Dengue-422657.aspx>. [Accessed and cited on 10 September 2024].
2. Vo LT, Nguyen DT, Tran TN, Tran HH, Đoan TT, Pham TN, et al. Pediatric Profound Dengue Shock Syndrome and Use of Point-of-Care Ultrasound During Mechanical Ventilation to Guide Treatment: Single-Center Retrospective Study, 2013-2021. Pediatr Crit Care Med. 2024; 25: e177-e185. doi: 10.1097/PCC.0000000000003413. Epub 2023 Nov 15. PMID: 37966344; PMCID: PMC10986784
