# Supplementary material for: Associations of resuscitation fluid load, colloid-to-crystalloid infusion ratio and clinical outcomes in children with dengue shock syndrome
Source: PLoS Negl Trop Dis. 2025 Jan 10;19(1):e0012786. doi: 10.1371/journal.pntd.0012786 (PMC11756768; doi:10.1371/journal.pntd.0012786)
Supplement: Attachment — Submitted filename: reviewed study.pdf [file pntd.0012786.s004.pdf]

PLOS Neglected Tropical Diseases

# Associations of resuscitation fluid load, colloid-to-crystalloid infusion ratio and clinical outcomes in children with dengue shock syndrome

--Manuscript Draft--

| | |
|---|---|
| **Manuscript Number:** | PNTD-D-24-01370 |
| **Full Title:** | Associations of resuscitation fluid load, colloid-to-crystalloid infusion ratio and clinical outcomes in children with dengue shock syndrome |
| **Short Title:** | Resuscitation fluid load, colloid-to-crystalloid ratio and dengue-associated outcomes |
| **Article Type:** | Research Article |
| **Keywords:** | Acute kidney injury; Colloid; Crystalloid; Colloid-to-crystalloid ratio, Dengue shock syndrome; Fluid resuscitation; Mechanical ventilation |
| **Abstract:** | Background<br>Severe respiratory distress and acute kidney injury (AKI) are key factors leading to poor outcomes in dengue shock syndrome (DSS). There ~~are~~ still limited data on how much resuscitated fluid and the specific ratios of intravenous fluid types contribute to the development of severe respiratory distress necessitating mechanical ventilation (MV) and AKI in children with DSS.<br>Methodology/Principal findings<br>This retrospective study was conducted at a tertiary pediatric hospital in Vietnam between 2013 and 2022. The primary outcomes were the need for MV and renal function within 48 h post-admission. A predictive model for MV was developed based on covariates from the first 24 h of PICU admission. Changes in renal function within 48 h were analyzed using a linear mixed-effects model. A total of 1,278 DSS children with complete clinical and fluid data were included. The predictive performance for MV based on the total intravenous fluid volume administered yielded an AUC of 0.871 (95% CI, 0.836–0.905), while the colloid-to-crystalloid ratio showed an AUC of 0.781 (95% CI, 0.743–0.819) (both $P < 0.001$). The optimal cut-off point of the cumulative fluid infusion was 181 mL/kg, whereas that of the colloid-to-crystalloid ratio was 1.6. Multivariable analysis identified female patients, severe bleeding, severe transaminitis, excessive fluid resuscitation, and a higher proportion of colloid solutions in the first 24 h as significant predictors of MV in DSS patients. The predictive model for MV demonstrated high accuracy, with a C-statistic of 89%, strong calibration, and low Brier score (0.04). Importantly, a more pronounced decline in glomerular filtration rate was observed in DSS patients who required MV than in those who did not.<br>Conclusions/Significance<br>This study provides insights into optimizing fluid management protocols, highlighting the importance of monitoring fluid volume and the colloid-to-crystalloid ratio during early resuscitation to improve the clinical outcomes of DSS patients. |
| **Additional Information:** | |
| **Question** | **Response** |
| **Government Employee**<br><br>Are you or any of the contributing authors an employee of the United States government?<br><br>Manuscripts authored by one or more US Government employees are not copyrighted, but are licensed under a CC0 Public Domain Dedication, which allows unlimited distribution and reuse of the | No - No authors are employees of the U.S. government. |

Powered by Editorial Manager® and ProduXion Manager® from Aries Systems Corporation

| | |
|---|---|
| article for any lawful purpose. This is a legal requirement for US Government employees.<br><br>This will be typeset if the manuscript is accepted for publication. | |
| **Financial Disclosure**<br><br>Enter a financial disclosure statement that describes the sources of funding for the work included in this submission. Review the submission guidelines for detailed requirements. View published research articles from *PLOS Neglected Tropical Diseases* for specific examples.<br><br>This statement is required for submission and **will appear in the published article** if the submission is accepted. Please make sure it is accurate.<br><br>**Funded studies**<br>Enter a statement with the following details:<br>• Initials of the authors who received each award<br>• Grant numbers awarded to each author<br>• The full name of each funder<br>• URL of each funder website<br>• Did the sponsors or funders play any role in the study design, data collection and analysis, decision to publish, or preparation of the manuscript?<br><br>Did you receive funding for this work? | The author(s) received no specific funding for this work. |
| **Competing Interests**<br><br>On behalf of all authors, disclose any competing interests that could be perceived to bias this work.<br><br>This statement will be typeset if the manuscript is accepted for publication.<br><br>Review the instructions link below and | All the authors declare no conflict of interest. |

| | |
|---|---|
| PLOS NTDs' competing interests policy to determine what information must be disclosed at submission. | |
| **Data Availability**<br><br>Provide a **Data Availability Statement** in the box below. This statement should detail where the data used in this submission can be accessed. This statement will be typeset if the manuscript is accepted for publication.<br><br>Before publication, authors are required to make all data underlying their findings fully available, without restriction. Review our PLOS Data Policy page for detailed information on this policy. Instructions for writing your Data Availability statement can be accessed via the Instructions link below. | The dataset of this study is available from corresponding author and is attached in the submission. |

*Powered by Editorial Manager® and ProduXion Manager® from Aries Systems Corporation*

Manuscript
Click here to access/download;Manuscript;Manuscript.docx

RESEARCH ARTICLE

# Associations of resuscitation fluid load, colloid-to-crystalloid infusion ratio and clinical outcomes in children with dengue shock syndrome

**Short title:** Resuscitation fluid load, colloid-to-crystalloid ratio and dengue-associated outcomes

**Authors:** Vo Thanh Luan [1 ¶], Vo Thi-Hong Tien [1], Ngo Thi-Mai Phuong [1], Do Chau Viet [1], Trinh Huu Tung [1], Nguyen Tat Thanh [1,2 ¶ *]

**Affiliations:**

[1] Department of Infectious Diseases, Children Hospital 2, Ho Chi Minh City, Vietnam

[2] TB Department, Woolcock Institute of Medical Research, Ho Chi Minh City, Vietnam

[¶] These authors contributed equally to this work.

[*] **Corresponding author:**

Nguyen Tat Thanh, MD PhD, TB Department, Woolcock Institute of Medical Research, Ho Chi Minh City, Vietnam. Email: thanhhonor@gmail.com / thanh.tat.nguyen@sydney.edu.au

ORCID number: 0000-0001-7600-6757

## Author summary

Dengue shock syndrome (DSS) in children admitted to the pediatric intensive care unit (PICU) has mortality rates ranging from 5% to > 20%, particularly in those with prolonged shock, severe respiratory distress requiring mechanical ventilation (MV), and acute kidney injury (AKI). Although colloidal solutions are commonly used in severe DSS management, excessive use can lead to respiratory failure and AKI, raising concerns regarding optimal fluid strategies. This study analyzed 1,278 children with DSS and found that 13.3% required MV, with a significantly higher mortality in the MV group (22.4%) than in the non-MV group (0.1%). Both the total intravenous fluid volume and colloid-to-crystalloid ratio were strong predictors of MV, with optimal cut-offs of 181 mL/kg for fluid volume and a ratio of 1.6. In practice, a cut-off of colloid-to-crystalloid ratio ≥ 1.6 may indicate the need to transition to alternative fluids such as albumin or fresh frozen plasma. Moreover, a marked reduction in the estimated glomerular filtration rate (eGFR) was observed in the MV group, indicating a link between fluid management and renal function. These findings underscore the importance of fluid management in DSS, providing key predictors of MV and AKI to guide treatment and improve clinical outcomes.

# Abstract

## Background

Severe respiratory distress and acute kidney injury (AKI) are key factors leading to poor outcomes in dengue shock syndrome (DSS). There ~~are~~ still limited data on how much resuscitated fluid and the specific ratios of intravenous fluid types contribute to the development of severe respiratory distress necessitating mechanical ventilation (MV) and AKI in children with DSS. is

## Methodology/Principal findings

This retrospective study was conducted at a tertiary pediatric hospital in Vietnam between 2013 and 2022. The primary outcomes were the need for MV and renal function within 48 h post-admission. A predictive model for MV was developed based on covariates from the first 24 h of PICU admission. Changes in renal function within 48 h were analyzed using a linear mixed-effects model. A total of 1,278 DSS children with complete clinical and fluid data were included. The predictive performance for MV based on the total intravenous fluid volume administered yielded an AUC of 0.871 (95% CI, 0.836–0.905), while the colloid-to-crystalloid ratio showed an AUC of 0.781 (95% CI, 0.743–0.819) (both $P < 0.001$). The optimal cut-off point of the cumulative fluid infusion was 181 mL/kg, whereas that of the colloid-to-crystalloid ratio was 1.6. Multivariable analysis identified female patients, severe bleeding, severe transaminitis, excessive fluid resuscitation, and a higher proportion of colloid solutions in the first 24 h as significant predictors of MV in DSS patients. The predictive model for MV demonstrated high accuracy, with a C-statistic of 89%, strong calibration, and low Brier score (0.04). Importantly, a more pronounced decline in glomerular filtration rate was observed in DSS patients who required MV than in those who did not.


The methodology section of abstract needs to be more concise like the way it has been in author summary

## Conclusions/Significance

This study provides insights into optimizing fluid management protocols, highlighting the importance of monitoring fluid volume and the colloid-to-crystalloid ratio during early resuscitation to improve the clinical outcomes of DSS patients.

## Keywords:

Acute kidney injury; Colloid; Crystalloid; Colloid-to-crystalloid ratio, Dengue shock syndrome; Fluid resuscitation; Mechanical ventilation

# Introduction

Dengue infection spans a spectrum from asymptomatic cases to fatalities, and dengue shock syndrome (DSS) stands out as the most common life-threatening dengue complications [1]. Patients with DSS admitted to the pediatric intensive care unit (PICU) have varied mortality rates ranging from 5% to 20% [1,2]. Mortality rates increase to > 30% in patients experiencing prolonged DSS, severe bleeding, acute liver failure, acute kidney injury (AKI), and severe respiratory failure requiring mechanical ventilation (MV) [3-6]. Notably, a cumulative intravenous fluid overload exceeding 10-15% is highly associated with respiratory failure and mortality in children with severe dengue cases [3-6]. This highlights the critical need for precise fluid resuscitation to reduce mortality in children with DSS. The World Health Organization (WHO) 2009 dengue guidelines recommend minimal fluid administration to ensure adequate tissue perfusion, using urine output > 0.5 ml/kg/h as an indicator for tapering fluid infusion [7]. However, to date, the optimal volume of resuscitated fluid and the appropriate colloid-to-crystalloid ratio for managing patients with DSS remain unclear.

complication

Colloidal solutions are often used to manage severe and prolonged DSS [8]. However, excessive colloid infusion can lead to AKI owing to lysosomal accumulation in the proximal tubules, resulting in tubular swelling and reduced renal blood flow pressure [9,10]. Furthermore, other critical factors such as severe bleeding, liver injury, and profound DSS can contribute to AKI risk but are not well documented. In particular, the effects of colloids on the dynamic variation of glomerular filtration rate (GFR) in DSS patients remain limited in the current literature [11-14].

A comprehensive understanding of fluid management strategies, particularly in DSS patients requiring MV, is essential for improving clinical outcomes. This study aimed to investigate the associations between resuscitation fluid load, the colloid-to-crystalloid infusion ratio, and

clinical outcomes in children with DSS admitted to the PICU. Our goal was to establish optimized fluid management protocols to minimize the need for MV and reduce the risk of kidney injury in pediatric DSS patients.

# Methods

## Data source and ethics statement

This sub-study stems from the main retrospective research titled "Prognostic model predicting mortality among patients presenting with dengue shock syndrome (DSS) at Children's Hospital 2, during 2013-2022," approved by the Scientific Committee and Institutional Review Board (IRB) of Children's Hospital 2, Ho Chi Minh City, Vietnam (IRB No. 893/QD-BVND2, signed on June 6, 2022) [15]. Using a secondary dataset from this primary research posed less than minimal risk to participants [15]. Therefore, the requirement for informed consent was waived, and patient identities were anonymized to ensure confidentiality in line with the ethical guidelines of the Declaration of Helsinki.

## Study setting and population

The primary study was conducted at the Children's Hospital 2, a tertiary pediatric hospital in southern Vietnam, spanning from 2013 to 2022 [15]. The eligibility criteria included children under 18 years of age with laboratory-confirmed dengue infection and clinical presentation of DSS, following the WHO 2009 dengue guidelines [7]. Exclusion criteria were the absence of serological confirmation of dengue infection and incomplete fluid resuscitation data.

## Study definitions

Dengue infection confirmation was based on the WHO 2009 criteria, relying on positive results for the non-structural 1 (NS1) antigen or Dengue-IgM antibody test [7]. Severe transaminitis was defined as elevated aspartate aminotransferase (AST) and/or alanine aminotransferase (ALT) levels ≥ 1,000 IU/L [7]. The definition of profound DSS involved two criteria: (i) the requirement of more than two colloid boluses during the first episode of compensated DSS or more than two episodes of recurrent shock, or (ii) the need for colloid resuscitation combined with inotropes to maintain stable hemodynamics [4,16]. Mechanical ventilation indications adhered to the Vietnamese Ministry of Health Dengue Guidelines, as outlined in **S1 File**.

## Study outcomes and candidate variables

The main study outcomes were mechanical ventilation requirement during the PICU stay and changes in renal function within 48 h after PICU admission. A predefined set of covariates was predetermined, including age, sex, DSS severity, severe bleeding, severe transaminitis, platelet counts, hematocrit levels, cumulative infused fluid volume within the first 24 h of PICU admission, and percentages of colloid and crystalloid fluids. These covariates were preselected based on our clinical knowledge and disease pathogenesis [2,4,15]. Notably, obesity data were missing in approximately 40% of the participants with available intravenous fluid infusion data; therefore, the obesity covariate was excluded from the analysis.

## Data collection and measurements

Clinical and laboratory data were collected at various time points and recorded in structured case report forms. The dataset included comprehensive information on age, sex, DSS severity, severe bleeding, liver transaminase levels, platelet counts, hematocrit levels, and the cumulative volume

of infused fluids during the first 24 h of PICU admission. Blood and chemical tests, including serum creatinine levels, were performed using an Alinity® ci-series machine (Abbott, USA). The estimated glomerular filtration rate (eGFR) was calculated using the Schwartz equation: estimated GFR = 36.5 × height (cm) / serum creatinine (µmol/L) [17]. Only the participants with complete datasets were included in the final analysis.

## Statistical analysis

Continuous variables were described using median and interquartile ranges (IQRs), while categorical variables were presented as numerical counts and percentages (%). The primary study bias was missing data on serum creatinine and patient height for eGFR estimation from retrospective data collection. To address this issue, only participants with complete data were included in the final analysis. The receiver operating characteristic (ROC) curve with area under the curve (AUC) values, sensitivity, specificity, and accuracy was evaluated to predict the risk of mechanical ventilation and changes in renal function within 48 h post-PICU admission. Univariate and multivariable logistic regression analyses were performed using complete-case analysis based on predefined sets of covariates chosen according to disease pathogenesis and clinical experience. The cumulative fluid volume from referral hospitals and within the first 24 h of PICU admission was log2-transformed for standardization. Additionally, the colloid-to-crystalloid infusion ratio was standardized using a square root transformation. Backward stepwise model selection, guided by the Akaike Information Criteria (AIC), was applied to determine the best predictive model, including interactions among covariates. Internal validation was performed using bootstrap resampling (n = 500). Model performance was assessed using the C-statistic, calibration slope, and Brier score [18,19]. Furthermore, a linear mixed-effects model was employed to explore the associations between total intravenous fluid load, colloid-to-crystalloid ratio, and renal function

changes within 72 h post-admission. All statistical tests were two-sided, with statistical significance set at $P < 0.05$. Data analyses were conducted using the R statistical software (version 4.3.2, Boston, MA, USA).

# Results

## Baseline characteristics of study participants upon PICU admission

Between 2013 and 2022, approximately 2,000 children with DSS were admitted to the PICU, of whom 1,278 met the eligibility criteria and were included in the analysis (**Fig1**). The clinical and laboratory data of the participants upon PICU admission are presented in **Table 1**. The median patient age was 8.1 years (interquartile range, IQR: 5.4 - 10.7), and females accounted for 48% of all patients. The median body mass index was 18.1 (IQR, 15.6 - 21.4) kg/m2, and 80 (6%) patients had underlying diseases. Participants experienced DSS on the median day 5 (IQR: 4–5 days) after the onset of fever. Notably, 1,171 (92%) children were diagnosed with compensated DSS and the remaining 107 (8%) patients had decompensated DSS. The systolic and diastolic shock indices were markedly elevated. Critical bleeding was observed in 95 (7.4%) patients. Complete blood counts showed a marked increase in hematocrit and decrease in platelet cell count. Severe transaminitis was observed in 130 (10.2%) participants. The median serum lactate was 2.3 (IQR, 1.7 - 3.3) mmol/L. Most notably, PICU-admitted patients with DSS received a high cumulative amount of intravenous (IV) fluids (crystalloid and colloid solutions) infused from referral hospitals and 24h PICU admission with a median of 135 (IQR, 105 - 181) mL/kg, and median ratio of colloid to crystalloid solution infused was 0.72 (IQR, 0 - 2.1). Additionally, 97 (7.6%) need vasopressors during the first 24h of admission, with a median vasoactive inotropic score (VIS) was 20 (IQR, 10 - 42.5).

[Fig 1 Caption]

**Fig 1. The flowchart of study participants**

**Table 1. Clinical and laboratory characteristics of study participants on PICU admission and associated clinical outcomes within 48h post-admission and at discharge (N = 1,278)**

| Characteristics | Summary statistics |
|---|---|
| ***On PICU admission*** | |
| Age (years) | 8.1 (5.4-10.7) |
| Sex: Female (%) | 619 (48) |
| Body mass index (kg/m$^2$) | 18.1 (15.6-21.4) |
| Underlying diseases (%) | 80 (6) |
| Day of DSS since disease onset (days) | 5 (4-5) |
| Grading of DSS severity (%) | |
| Compensated DSS | 1,171 (92) |
| Decompensated DSS | 107 (8) |
| Severe bleeding (%) | 95 (7.4) |
| Respiratory rate (/min) | 25 (22-30) |
| Systolic shock index (bpm/mmHg) | 1.30 (1.11-1.5) |
| Diastolic shock index (bpm/mmHg) | 1.69 (1.44-2.0) |
| White blood cell count (x 10$^9$/L) | 4.8 (3.37-6.8) |
| Hemoglobin (g/dL) | 14.9 (13.4-16.1) |
| Peak hematocrit (%) | 48 (45-51) |
| Nadir hematocrit (%) | 38 (35-41) |
| Platelet counts (x 10$^9$/L) | 36 (23-55) |
| Aspartate aminotransferase (IU/L) | 151 (87-346) |
| Alanine aminotransferase (IU/L) | 66 (35-169) |
| Severe transaminitis (%) | 130 (10.2) |
| International normalized ratio (INR) | 1.23 (1.1-1.49) |
| Troponin I (ng/mL) | 0.012 (0.01-0.054) |
| Serum lactate (mmol/L) | 2.3 (1.7-3.3) |
| Arterial blood gas analysis | |
| pH | 7.44 (7.40-7.48) |
| $PCO_2$ (mmHg) | 26.1 (22-30.2) |
| $PO_2$ (mmHg) | 111 (65-149) |
| Bicarbonate (mEq/L) | 17.2 (14.7-19.7) |
| Cumulative fluid infused from referral hospitals and 24h PICU admission (mL/kg) | 135 (105-181) |
| Ratio of colloid-to-crystalloid infusion | 0.72 (0 - 2.1) |
| Vasopressor support during first 24h (%) | 97 (7.6) |
| Vasoactive inotropic score | 20 (10-42.5) |

| Clinical outcomes of patients | |
|---|---|
| ***Within first 48h of PICU admission*** | |
| Mechanical ventilation support (%) | 170 (13.3) |
| eGFR on admission (mL/min) | 86 (72-100) |
| eGFR at 48h post-admission (mL/min) | 94 (76-108) |
| ***At hospital discharge*** | |
| Length of hospital stay (days) | 4 (3-6) |
| Fatal outcome (%) | 39 (3.1) |

Summary statistics are presented as median (interquartile range, IQR) for continuous variables and frequency (%) for categorical variables. **Abbreviations:** DSS, dengue shock syndrome; eGFR, estimated glomerular filtration rate by Schwartz equation; PICU, pediatric intensive care unit

## Clinical outcomes of study participants

On admission, patients exhibited a slight increase in respiratory rate (median 25 breaths/min; IQR, 22-30) and mild abnormalities in blood gas analysis. Despite this, 170 (13.3%) progressed to severe respiratory failure, requiring mechanical ventilation within 48 h of PICU admission (**Table 1**). The estimated glomerular filtration rate (eGFR) showed a slight improvement at 48 h post-admission (median 86 mL/min; IQR, 72 - 100 mL/min) compared to admission values (median 94 mL/min; IQR, 76 - 108 mL/min).

Overall, 39 of 1,278 (3.1%) DSS patients died during the PICU stay, with a median hospital stay of 4 days (IQR, 3-6). Among those requiring mechanical ventilation, the mortality rate was significantly higher (22.4%). The primary causes of death included acute respiratory distress syndrome (ARDS), pulmonary and cerebral hemorrhage, dengue-associated acute liver failure, multiorgan failure, and severe PICU-acquired infections.

## Predicting the risk of mechanical ventilation, based on fluid resuscitation data during the first 24 h of admission

The performance of prognostic indicators for predicting mechanical ventilation (MV) in DSS children is summarized in **Fig 2** and **Table 2**. A large volume of intravenous fluids infused from referral hospitals and during the first 24 hours of PICU admission was a strong predictor of MV, with an AUC of 0.871 (95% CI, 0.836-0.905, $P < 0.001$). A higher colloid-to-crystalloid infusion ratio was also a significant predictor with an AUC of 0.781 (95% CI, 0.743-0.819, $P < 0.001$). The blood products used in DSS patients with critical bleeding, low platelet counts, and severe plasma leakage demonstrated a high predictive value for MV. Further analysis identified the optimal cut-off points for these indicators. A total IV fluid infusion of ≥ 181 mL/kg had a sensitivity of 78.2% and a specificity of 82.8% for predicting the requirement for MV support. The cut-off for colloid-to-crystalloid infusion ratio was ≥ 1.6, with a sensitivity of 68.8% and a specificity of 73.5%. These findings underscore the importance of fluid management strategies in early DSS care to identify patients at risk for MV.

[Fig 2 Caption]

**Fig 2. Area under the receiver operating characteristic curves (AUCs) for the cumulative amount of intravenous fluid infusion and colloid-to-crystalloid ratio in predicting the need for MV**.

**Table 2. Area under the curve for cumulative infused fluid from referral hospitals and during the first 24h of admission and cutoffs for mechanical ventilation**

| **Parameters** | **AUC of MV support (95% confidence interval)** | | ***P*-value** |
|---|---|---|---|
| Cumulative IV fluid infused from referral hospitals and 24h PICU admission (mL/kg) | 0.871 | 0.836-0.905 | < 0.001 |

| | | | |
|---|---|---|---|
| Colloid fluid (%) | 0.701 | 0.664-0.739 | < 0.001 |
| Crystalloid fluid (%) | 0.842 | 0.811-0.872 | < 0.001 |
| Blood products (%) [a] | 0.900 | 0.870-0.930 | < 0.001 |
| Ratio of colloid-to-crystalloid infusion | 0.781 | 0.743-0.819 | < 0.001 |
| **Optimal cutoff points** | **Sensitivity** | **Specificity** | **Accuracy** |
| Total volume of fluid infusion ≥ 181(mL/kg) | 0.782 | 0.828 | 0.822 |
| Ratio of colloid-to-crystalloid infusion ≥ 1.6 | 0.688 | 0.735 | 0.729 |

**Abbreviation:** AUC, Area under the curve; MV, Mechanical ventilation; PICU, Pediatric intensive care unit

[a] Blood products included packed red blood cells, platelet transfusion, fresh frozen plasma, and albumin solutions

## Associations of resuscitation fluid load, colloid-to-crystalloid infusion ratio and the need for MV in children with DSS

As shown in **Table 3**, multivariable backward stepwise logistic regression analysis using AIC values identified key prognostic indicators for MV requirement in DSS patients. Significant factors included female patients, severe bleeding, severe transaminitis, a large cumulative amount of fluid infusion from referral hospitals and within the first 24 h of PICU admission, and a high colloid-to-crystalloid infusion ratio. Interestingly, a high hematocrit level served as a protective factor. No significant interactions among the covariates were detected.

**Table 3. Associations of resuscitation fluid load, colloid-to-crystalloid ratio and the need for mechanical ventilation in children with DSS**

| **Risk factors** | **MV (n = 170)** | **Non-MV (n =1,108)** | **Unadjusted effect** | **Adjusted effect** |
|---|---|---|---|---|
| | **Summary statistic** | **Summary statistic** | **OR (95% CI), *P*-value** | **OR (95% CI), *P*-value** |
| Age (years) | 6.9 (4-9) | 8.4 (5.7-10.9) | 0.89 (0.85-0.93), *P* < .001 | - |

| | | | | |
|---|---|---|---|---|
| Patient sex (%) | | | | |
| Male | 75 (44) | 584 (53) | 1.42 (1.03-1.98), $P = .038$ | 1.88 (1.11-3.18), $P = .02$ |
| Female | 95 (56) | 524 (47) | | |
| Severe bleeding (%) [a] | | | | |
| No | 93 (55) | 1090 (98) | 56.7 (31.8-101), $P < .001$ | 20.74 (8.77-49.1), $P < .001$ |
| Yes | 77 (45) | 18 (2) | | |
| Severe transaminitis (%) [a] | | | | |
| No | 102 (60) | 1046 (94) | 11.1 (7.41-16.63), $P < .001$ | 6.21 (3.16-12.2), $P < .001$ |
| Yes | 68 (40) | 62 (6) | | |
| Hematocrit (%) | 47 (41-50) | 48 (45-51) | 0.91 (0.88-0.93), $P < .001$ | 0.89 (0.84-0.93), $P < .001$ |
| Platelet counts (< 20 x $10^9$/L), (%) | | | | |
| No | 120 (71) | 900 (82) | 1.87 (1.3-2.68), $P < .001$ | - |
| Yes | 50 (29) | 201 (18) | | |
| *Log2*-Cumulative infused fluid from referral hospitals and 24h PICU admission (mL/kg) [b] | 8.01 (7.55-8.48) | 6.99 (6.66-7.35) | 17.7 (11.78-26.62), $P < .001$ | 14.18 (8.1-24.96), $P < .001$ |
| *Square root*-Ratio of colloid-to-crystalloid infusion [c] | 1.76 (1.04-2.63) | 0.75 (0-1.31) | 3.45 (2.83-4.22), $P < .001$ | 2.2 (1.55-3.1), $P < .001$ |
| **Performance of multivariable model developed: internal validation [d]** | | | | |
| C-statistic | 0.89 (0.88) | | | |
| Calibration-in-the-large | 0 (- 0.03) | | | |
| Calibration slope | 1 (0.97) | | | |
| Brier score | 0.04 (0.04) | | | |

Summary statistics are median (IQR) for continuous variables and frequency (%) for categorical data. **Abbreviations**: CI, Confidence interval; DSS, Dengue shock syndrome; OR, Odds ratio, PICU, Pediatric intensive care unit.

[a] These factors were defined by the 2009 WHO dengue guidelines

[b] Standardized covariate of the cumulative amount of intravenous fluid from referral hospitals and within 24h PICU admission by log2-transformation.

[c] Standardized covariate of the ratio of colloid-to-crystalloid infusion by square root

[d] Internal validation was performed in training (test) sets with bootstrap (n = 500)

## Performance of predictive model for mechanical ventilation and internal validation

The predictive model for mechanical ventilation in DSS patients exhibited strong performance (**Table 3**). It demonstrated high discrimination with a C-statistic of 89%, along with good calibration and low Brier scores in both training and test sets. The calibration plot (**Fig 3**) showed high agreement between the predicted and observed outcomes, indicating the reliability of the model for predicting mechanical ventilation in this patient population.

[Fig 3 Caption]

**Fig 3. Calibration plot for the predictive model of mechanical ventilation in patients with dengue shock syndrome.** The developed prognostic model showed good consistency between the predicted values (x-axis) and observed data (y-axis).

## Changes in the estimated glomerular filtration rate by mechanical ventilation upon PICU admission and 48h post-admission

The dynamic variations in renal function at admission and 48 h post-admission in MV and non-MV patients are shown in **Fig. 4A**. There was a significant difference in renal function between DSS patients requiring MV and those who did not. In the non-MV group, eGFR improved, whereas in the MV group, it deteriorated. Further analysis revealed a significant decline in eGFR among MV patients, from a mean of 81 mL/min on admission to 73 mL/min 48 h after PICU admission ($P$ = 0.01, paired t-test). Conversely, DSS patients not requiring MV showed a significant improvement in eGFR, increasing from 92 mL/min on admission to 103 mL/min at 48 h after hospitalization ($P$ < 0.001, paired t-test; **Fig 4B**). These findings suggest that DSS patients

undergoing MV are more prone to kidney injury, as indicated by reduced eGFR, than those who do not require MV.

[Fig 4 Caption]

**Fig 4. Dynamic changes in estimated glomerular filtration rates between the MV and non-MV groups at PICU admission and 48 h post-admission.**

## Associations of within 48h-admission dynamic changes in glomerular filtration rate, and resuscitation fluid load

As shown in **Table 4**, the linear mixed-effects model demonstrated dynamic changes in glomerular filtration rate (eGFR) from PICU admission to 48 hours post-admission in DSS patients. Random effect analysis revealed an intra-group correlation of 5%, indicating that the mechanical ventilation factor contributed to 5% of the variance in eGFR. The fixed effects model showed that a high cumulative amount of colloid infusion was significantly associated with eGFR reduction in DSS patients. However, the colloid-to-crystalloid infusion ratio did not show a statistically significant correlation. Notably, profound DSS, severe bleeding, and severe transaminitis were identified as significant prognostic indicators of decreased eGFR. Further analysis showed good normality of the residual parameter from the linear mixed model, which indicated the high validity of the developed model.

**Table 4. Linear mixed effect model of for changes in glomerular filtration rate on PICU admission and 48h post-admission in patients with DSS (n = 377)**

| **Random effect analysis** | **Variance** | **Standard deviation** | | |
|---|---|---|---|---|
| Groups (MV and Non-MV) | | | | |
| Intercept | 39 | 6.2 | | |
| Residuals [a] | 721 | 27 | | |
| **Fixed effect analysis** | **Estimate** | **SE** | **95% CI** | ***P*- value** |
| Intercept | 90.3 | 5.0 | 80.5 to 100.1 | 0.01 |
| Square root of cumulative amount of colloid fluid infused during 24h admission (mL/kg) [b] | 0.84 | 0.39 | 0.08 to 1.60 | 0.03 |
| Square root of ratio of colloid-to-crystalloid fluid infusion [b] | - 2.64 | 1.97 | -6.5 to 1.22 | 0.18 |
| Profound DSS | - 15.24 | 3.44 | -22 to -8.5 | < 0.001 |
| Severe bleeding | - 7.94 | 3.10 | -14 to -1.86 | 0.01 |
| Severe transaminitis | - 6.86 | 3.02 | -12.8 to 0.94 | 0.02 |

**Abbreviations:** CI, 95% confidence interval; DSS, Dengue shock syndrome; MV, mechanical ventilation; PICU, Pediatric intensive care unit; SE, Standard error

[a] Random effect was adjusted for mechanical ventilation

[b] Standardisation of covariates by the square root transformation

# Discussion

The fatality rate of patients with dengue shock syndrome ranges from 5% to 20% [1,2]. In particular, a dramatically higher in-hospital mortality rate (> 30%) has been observed in DSS patients with prolonged shock, dengue-associated acute liver failure, and severe respiratory failure requiring mechanical ventilation [3-5]. Although colloidal solutions are recommended for treating patients with severe and prolonged DSS [8], an excessive amount of intravenous (IV) fluid infusion, particularly colloidal solutions, is strongly associated with adverse outcomes among patients with DSS [3,6,9,10]. Therefore, we investigated the associations and prognostic values of

resuscitation fluid load and the percentages of colloid and crystalloid solutions, particularly the colloid-to-crystalloid infusion ratio, in relation to clinical outcomes, such as MV requirement and reduced renal function, in children with DSS admitted to the PICU.

In this study, 13.3% of the patients with DSS developed severe respiratory failure requiring MV within 48 h of PICU admission, and the overall in-hospital mortality rate was 3.1%. Critically, the fatality rate among patients with severe and prolonged DSS who required MV was as high as 22.4%. This cohort exhibited salient features, including profound DSS with progressively massive plasma leakage and elevated intra-abdominal and thoracic pressures [2,4]. Such conditions lead to significant preload reduction, necessitating large volumes of resuscitation fluids, ultimately contributing to multiorgan damage and death [4,20]. Resuscitation using macromolecular solutions is associated with increased mortality and acute kidney injury (AKI), whereas improved outcomes have been reported in patients treated with hyperoncotic albumin [21].

Our study highlighted notable findings considering that the patient cohort experiencied prolonged DSS and required MV. This group was resuscitated with large volumes of colloid solutions, resulting in a higher colloid-to-crystalloid ratio than that in the non-MV group (**Table 3**). Our study shows that total cumulative fluid infusion within the first 24 h of PICU admission is a strong predictor of mechanical ventilation, which is consistent with previous reports [6,22,23]. Importantly, administering macromolecular solutions to severe DSS patients with extensive plasma leakage, who do not respond to initial crystalloid resuscitation, is often inevitable. However, the high volume of colloid solution poses a significant risk of severe respiratory failure, necessitating mechanical ventilation. Specifically, our study found that a colloid-to-crystalloid ratio cut-off point of $\geq$ 1.6 yielded a high predictive value with an AUC of 78.1% for MV requirement. These results underscore several critical findings. First, large volumes of

macromolecular solutions administered to patients with severe DSS for many consecutive hours were due to progressively extensive plasma leakage. Second, in patients with prolonged and recurrent dengue shock, early fluid resuscitation with a large colloid volume significantly correlated with mechanical ventilation support. Lastly, inappropriate and prolonged use of colloid solutions beyond the actual demand increases the risk of MV in DSS patients.

Our study also yields a marked finding that rising hematocrit was a protective factor against the need for MV (**Table 3**). This indicates that restricting IV fluids is recommended, despite the relatively elevated hematocrit levels. Therefore, the use of colloidal solutions should be minimized to ensure adequate hemoperfusion. Additionally, a portion of colloid fluids should be replaced early with hyperoncotic albumin or fresh frozen plasma in cases of severe bleeding and coagulation disorders. A retrospective study by Lalitha *et al.* on 76 children with DSS showed that 20% albumin rapidly decreased lactate levels and shortened the duration of mechanical ventilation and hospital stay compared to 5% albumin [24]. Notably, albumin potentially protects and maintains the integrity of the endothelial glycocalyx layer, reducing the rate of plasma leakage into interstitial tissues, and restoring microcirculatory hemoperfusion [25]. In particular, concentrated albumin helps reabsorb fluid from the interstitial tissue, restore circulatory volume, and reduce fluid demand [26,27].

To the best of our knowledge, this is the first study to examine the colloid-to-crystalloid ratio to predict the risk of mechanical ventilation in pediatric patients with DSS. When fluid resuscitation with colloid solutions is mandatory for patients with severely prolonged DSS, restricting the amount of colloid fluid can be challenging. This is particularly true when patients experiencing hypotensive shock receive high doses of colloids soon after the initial failure of crystalloid fluid resuscitation [7]. In this context, the colloid-to-crystalloid ratio serves as a

significant indicator for guiding colloid fluid resuscitation and suggests the optimal time to substitute colloid solutions with other beneficial fluids, such as hyperoncotic albumin. Additionally, colloid solutions potentially cause coagulation disorders and pose a higher risk of bleeding, which may necessitate more blood product transfusions [28]. Excessive hemodilution from large amounts of colloid solutions can paradoxically decrease oxygen delivery (DO2) by reducing hemoglobin levels, complicating the identification of severe bleeding, and potentially leading to unnecessary transfusions [29]. This can increase the risk of mechanical ventilation and mortality owing to fluid overload. Our results also indicated that more blood product transfusions were associated with MV support, possibly correlating with the overuse of macromolecular solutions.

Importantly, it has been reported that approximately 4% of hospitalized patients with severe dengue infection develop acute kidney injury (AKI), with 14.1% requiring continuous renal replacement therapy for critical AKI [12]. In this study, 164 of 1,278 participants (12.8%) experienced severe, prolonged, and recurrent dengue shock, necessitating high doses of colloid fluid resuscitation. However, excessive use of colloidal fluids may increase the risk of AKI and coagulation disorders [30,31]. Clinical data on AKI due to colloid overuse in patients with severe DSS undergoing MV remain limited. Therefore, we aimed to explore the impact of high colloid fluid infusion on renal function within 48 h of PICU admission in patients with profound DSS with and without MV support. Notably, our study also included severe DSS patients referred from provincial hospitals who had already received large volumes of IV fluid, complicated by severe respiratory failure. The most important finding of this study is that children with DSS who experienced severe respiratory failure requiring mechanical ventilation were more likely to have acute kidney injury, as indicated by decreased eGFR, compared to those who did not require MV

support. Conversely, DSS patients without MV support responded well to fluid resuscitation and recovered quickly, as evidenced by the rapid improvement in kidney function 48 h post-PICU admission. One plausible explanation is that compared to DSS patients without MV support, those with MV received a substantially higher volume of resuscitation fluids, including a higher percentage of colloids (**Table 3**). Another important point is that the random effects revealed that only a 5% difference in eGFR was explained by mechanical ventilation. This suggests that other determinants from fixed-effect modeling, including severe bleeding, severe transaminitis, and severe, prolonged DSS, have played a more prominent role. These factors are crucial in the pathogenesis of severe dengue and serve as significant predictors of MV in children with DSS [2,4,6]. Our study findings on the prognostic factors for AKI in severe dengue patients are consistent with those of previous reports [11-14]. Possible causes of AKI in children with DSS include (i) pre-renal factors such as intravascular volume inadequacy, hypovolemia, and reduced hemoperfusion to the kidneys due to severe bleeding and progressive vascular leakage in severe DSS patients, and (ii) at-renal indicators such as proximal tubule injury and damage caused by the high load of colloid solutions administered to severe and prolonged DSS patients [9,10]. Both Wang *et al*. and Diptyanusa *et al.* reported significant case fatality rates in DSS patients with AKI requiring dialysis, ranging from approximately 14% to 22%. In contrast, most patients in our study with severe DSS and AKI survived at hospital discharge. The major causes of DSS in our study were acute respiratory distress syndrome (ARDS), pulmonary and cerebral hemorrhage, dengue-associated acute liver failure, and multiorgan failure [4,5].

Remarkably, our research group recently reported that the cumulative amount of infused fluid is a significant predictor of in-hospital mortality in children with DSS [15]. In this study, we further identified that a large cumulative amount of intravenous fluid infusion ($\geq$ 181 mL/kg),

particularly with a high proportion of colloid solutions, was associated with adverse dengue-related complications including mechanical ventilation requirement and acute kidney injury. Notably, higher fatality rates have been reported in patients with dengue who experience acute respiratory failure and AKI [3,13]. In clinical practice, a small proportion of patients with severe and prolonged DSS experience progressively massive plasma leakage, leading to a substantial increase in thoracic pressure and the need for MV [4,20]. In such cases, an intensive fluid intervention scheme with hemodynamic monitoring, preferably under point-of-care ultrasound (POCUS) guidance, is recommended. A higher volume of resuscitation fluid has been shown to yield better survival outcomes based on the improvement in serum lactate levels, vasoactive inotropic score, and organ dysfunction [4]. However, this approach should be cautiously considered, as most patients in that study cohort were in the very late stage of recurrent and/or profound DSS with extensive vascular leakage and preload insufficiency [4]. For the general population with dengue shock syndrome, minimal fluid administration to ensure tissue perfusion remains the cornerstone of dengue treatment, in line with the WHO 2009 dengue guidelines [7]. Additional resuscitation fluid should only be administered to confer a survival benefit in a small subset of patients experiencing dengue-associated obstructive shock syndrome [4,20]. Further comprehensive studies focusing on this critical population are needed to provide more convincing evidence in this regard.

Our study had several limitations inherent to its single-center, retrospective cohort design and non-standardized collection of clinical and laboratory data during hospital admission. A significant potential source of information bias arises from differences in disease severity assessment and fluid management between patients referred from provincial hospitals (who had already received resuscitation fluids) and those directly admitted to our hospital. The referred

group commonly presented with more severe dengue, such as prolonged and severe DSS, compared to the direct admission group. Despite these limitations, our study population was relatively representative and generalizable. Further investigation in prospective cohorts with appropriate study designs is essential to validate our findings.

# Conclusion

In conclusion, a significant volume of resuscitation fluid (≥ 181 mL/kg) and a higher colloid-to-crystalloid infusion ratio (≥ 1.6) were associated with adverse outcomes, including the need for mechanical ventilation and acute kidney injury within the first 48 h of admission to the PICU. Other critical prognostic factors included profound dengue shock, severe transaminitis, and critical bleeding. These findings are essential for optimizing fluid management protocols to enhance the clinical outcomes in patients with dengue shock syndrome.

## Abbreviations

Acute kidney injury (AKI), Dengue shock syndrome (DSS), estimated glomerular filtration rate (eGFR), Mechanical ventilation (MV), Pediatric intensive care unit (PICU)

# Supporting information

**S1 Checklist. STROBE checklist** (DOCX)

**S1 File. Indications for mechanical ventilation support in children with severe DSS** (DOCX)

**S1 Appendix**. **Observational data of 1,278 participants in the study cohort** (ZIP)

## Acknowledgements

We are grateful to the patients and administrative staffs, particularly Drs DTT Hien and TTTri for their support with this study.

### Availability of data and materials

The dataset of this study is available from corresponding author on legitimate request.

### Funding statement

This study was self-funded.

### Consent for publication

All the authors approved with this final manuscript submitted for publication.

### Competing Interests

All the authors declare no conflict of interest.

## Author Contributions

All authors have contributed to and approved the final manuscript.

**Conceptualization:** Thanh NT, Luan VT, Tung TH

**Data curation:** Thanh NT, Luan VT, Tien VTH, Phuong NTM

**Formal analysis:** Thanh NT

**Funding acquisition:** Luan VT, Viet DC

**Investigation**: Thanh NT, Luan VT, Luan VT, Tien VTH, Phuong NTM , Viet DC

**Methodology:** Thanh NT, Luan VT

**Project administration:** Tung TH, Luan VT, Viet DC

**Resources:** Luan VT, Viet DC, Tung TH

**Supervision:** Luan VT, Viet DC, Tung TH

**Writing - original draft:** Thanh NT, Luan VT

**Critical revision of the final manuscript**: Thanh NT, Viet DC, Tung TH, Luan VT

# References


1. World Health Organization. Global strategy for dengue prevention and control 2012-2020. World Health Organization, 2012.
2. Nguyen TT, Nguyen DT, Vo TT, Dang OT, Nguyen BT, Pham DT, et al. Associations of obesity and dengue-associated mortality, acute liver failure and mechanical ventilation in children with dengue shock syndrome. Medicine (Baltimore). 2023; 102: e36054. doi: 10.1097/MD.0000000000036054. PMID: 37986332; PMCID: PMC10659721.
3. Preeprem N, Phumeetham S. Paediatric dengue shock syndrome and acute respiratory failure: a single-centre retrospective study. BMJ Paediatr Open. 2022; 6: e001578. doi: 10.1136/bmjpo-2022-001578. PMID: 36645744; PMCID: PMC9668030.
4. Vo LT, Nguyen DT, Tran TN, Tran HH, Đoan TT, Pham TN, et al. Pediatric Profound Dengue Shock Syndrome and Use of Point-of-Care Ultrasound During Mechanical Ventilation to Guide Treatment: Single-Center Retrospective Study, 2013-2021. Pediatr Crit Care Med. 2024; 25: e177-e185. doi: 10.1097/PCC.0000000000003413. Epub 2023 Nov 15. PMID: 37966344; PMCID: PMC10986784.
5. Thanh NT, Dat NT, Thinh TN, Phuong NTM, Thanh MTH, Bao NT, et al. Therapeutic plasma exchange and continuous renal replacement therapy in pediatric dengue-associated acute liver

failure: A case series from Vietnam. Transfus Apher Sci. 2023; 62: 103617. doi: 10.1016/j.transci.2022.103617. Epub 2022 Nov 28. PMID: 36522271.

6. Sachdev A, Pathak D, Gupta N, Simalti A, Gupta D, Gupta S, et al. Early Predictors of Mortality in Children with Severe Dengue Fever: A Prospective Study. Pediatr Infect Dis J. 2021; 40 (9): 797-801. doi: 10.1097/INF.0000000000003179. PMID: 34321449
7. WHO. Dengue: Guidelines for Diagnosis, Treatment, Prevention and Control. Geneva: World Health Organization, 2009. Available from: https://iris.who.int/handle/10665/44188
8. Wills BA, Nguyen MD, Ha TL, Dong TH, Tran TN, Le TT, et al. Comparison of three fluid solutions for resuscitation in dengue shock syndrome. N Engl J Med. 2005; 353: 877-89. doi: 10.1056/NEJMoa044057. PMID: 16135832.
9. Dickenmann M, Oettl T, Mihatsch MJ. Osmotic nephrosis: acute kidney injury with accumulation of proximal tubular lysosomes due to administration of exogenous solutes. Am J Kidney Dis. 2008; 51: 491-503. doi: 10.1053/j.ajkd.2007.10.044. PMID: 18295066.
10. Schortgen F, Girou E, Deye N, Brochard L; CRYCO Study Group. The risk associated with hyperoncotic colloids in patients with shock. Intensive Care Med. 2008; 34: 2157-68. doi: 10.1007/s00134-008-1225-2. Epub 2008 Aug 7. PMID: 18685828.
11. Khalil MA, Sarwar S, Chaudry MA, Maqbool B, Khalil Z, Tan J, et al. Acute kidney injury in dengue virus infection. Clin Kidney J. 2012; 5: 390-4. doi: 10.1093/ckj/sfs117. PMID: 26019813; PMCID: PMC4432424.
12. Diptyanusa A, Phumratanaprapin W, Phonrat B, Poovorawan K, Hanboonkunupakarn B, Sriboonvorakul N, et al. Characteristics and associated factors of acute kidney injury among adult dengue patients: A retrospective single-center study. PLoS One. 2019; 14: e0210360. doi: 10.1371/journal.pone.0210360. PMID: 30615667; PMCID: PMC6322747.

13. Huy BV, Thuy DT. Prevalence, Characteristics, and Factors Associated with Acute Kidney Injury among Adult Dengue Patients in Vietnam. Am J Trop Med Hyg. 2020; 104: 1067-1071. doi: 10.4269/ajtmh.20-0840. PMID: 33319734; PMCID: PMC7941826.

14. Wang C, Hong W, Ou Z, Yang H, Zhao L, Zhang Z, et al. Prevalence, Characteristics, and Outcomes Associated with Acute Kidney Injury among Adult Patients with Severe Dengue in Mainland China. Am J Trop Med Hyg. 2023; 109: 404-412. doi: 10.4269/ajtmh.22-0803. PMID: 37364862; PMCID: PMC10397446.

15. Nguyen Tat T, Vo Hoang-Thien N, Nguyen Tat D, Nguyen PH, Ho LT, Doan DH, et al. Prognostic values of serum lactate-to-bicarbonate ratio and lactate for predicting 28-day in-hospital mortality in children with dengue shock syndrome. Medicine (Baltimore). 2024; 103: e38000. doi: 10.1097/MD.0000000000038000. PMID: 38669370; PMCID: PMC11049702.

16. Lam PK, Tam DT, Dung NM, Tien NT, Kieu NT, Simmons C, et al. A Prognostic Model for Development of Profound Shock among Children Presenting with Dengue Shock Syndrome. PLoS One. 2015; 10: e0126134. doi: 10.1371/journal.pone.0126134. PMID: 25946113; PMCID: PMC4422752.

17. Selistre L, Rabilloud M, Cochat P, de Souza V, Iwaz J, Lemoine S, et al. Comparison of the Schwartz and CKD-EPI Equations for Estimating Glomerular Filtration Rate in Children, Adolescents, and Adults: A Retrospective Cross-Sectional Study. PLoS Med. 2016; 13: e1001979. doi: 10.1371/journal.pmed.1001979. PMID: 27023756; PMCID: PMC4811544.

18. Steyerberg EW, Vickers AJ, Cook NR, Gerds T, Gonen M, Obuchowski N, et al. Assessing the performance of prediction models: a framework for traditional and novel measures. Epidemiology. 2010; 21:128-38. doi: 10.1097/EDE.0b013e3181c30fb2. PMID: 20010215; PMCID: PMC3575184.

19. Harrell F. Regression Modeling Strategies with Applications to Linear Models, Logistic and Ordinal Regression, and Survival Analysis. Srpinger New York 2015.
20. Nguyen TT, Le NT, Nguyen NM, Do VC, Trinh TH, Vo LT. Clinical features and management of children with dengue-associated obstructive shock syndrome: A case report. Medicine (Baltimore). 2022; 101: e31322. doi: 10.1097/MD.0000000000031322. PMID: 36316901; PMCID: PMC9622697.
21. Wiedermann CJ, Dunzendorfer S, Gaioni LU, Zaraca F, Joannidis M. Hyperoncotic colloids and acute kidney injury: a meta-analysis of randomized trials. Crit Care. 2010;14: R191. doi: 10.1186/cc9308. Epub 2010 Oct 28. PMID: 21029460; PMCID: PMC3219298.
22. Raina R, Sethi SK, Wadhwani N, Vemuganti M, Krishnappa V, Bansal SB. Fluid Overload in Critically Ill Children. Front Pediatr. 2018; 6:306. doi: 10.3389/fped.2018.00306. PMID: 30420946; PMCID: PMC6215821.
23. Alobaidi R, Morgan C, Basu RK, Stenson E, Featherstone R, Majumdar SR, et al. Association Between Fluid Balance and Outcomes in Critically Ill Children: A Systematic Review and Meta-analysis. JAMA Pediatr. 2018; 172: 257-268. doi: 10.1001/jamapediatrics.2017.4540. PMID: 29356810; PMCID: PMC5885847.
24. Lalitha, A.V. Impact of resuscitation with 20% versus 5% albumin in outcome of severe dengue: a prospective observational study. Pediatric Critical Care Medicine. 2018; 19: p 36. [DOI: 10.1097/01.pcc.0000537423.59159.10]
25. Aldecoa C, Llau JV, Nuvials X, Artigas A. Role of albumin in the preservation of endothelial glycocalyx integrity and the microcirculation: a review. Ann Intensive Care. 2020; 10: 85. doi: 10.1186/s13613-020-00697-1. PMID: 32572647; PMCID: PMC7310051.

26. Zdolsek M, Hahn RG, Zdolsek JH. Recruitment of extravascular fluid by hyperoncotic albumin. Acta Anaesthesiol Scand. 2018; 62: 1255-1260. doi: 10.1111/aas.13150. Epub 2018 May 29. PMID: 29845612.
27. Haynes, G.R., Bassiri, K. Hyper-oncotic vs. Hypo-oncotic Albumin Solutions: a Systematic Review of Clinical Efficacy and Safety. SN Compr Clin Med. 2021; 3: 1137–47. [Available from: https://doi.org/10.1007/s42399-021-00755-0].
28. Haase N, Perner A, Hennings LI, Siegemund M, Lauridsen B, Wetterslev M, et al. Hydroxyethyl starch 130/0.38-0.45 versus crystalloid or albumin in patients with sepsis: systematic review with meta-analysis and trial sequential analysis. BMJ. 2013; 346:f839. doi: 10.1136/bmj.f839. PMID: 23418281; PMCID: PMC3573769.
29. Perel A. Iatrogenic hemodilution: a possible cause for avoidable blood transfusions? Crit Care 2017; 21: 291. [ PMID: 29178938; PMCID: PMC5702064; DOI: 10.1186/s13054-017-1872-1]
30. Hung NT. Fluid management for dengue in children. Paediatr Int Child Health. 2012; 32 Suppl 1(s1):39-42. doi: 10.1179/2046904712Z.00000000051. PMID: 22668449; PMCID: PMC3381450.
31. Mailloux L, Swartz CD, Capizzi R, Kim KE, Onesti G, Ramirez O, et al. Acute renal failure after administration of low-molecular-weight dextran. N Engl J Med. 1967; 277: 1113-8. doi: 10.1056/NEJM196711232772103. PMID: 6054998.

Fig1

Click here to access/download;Figure;Fig1.tif

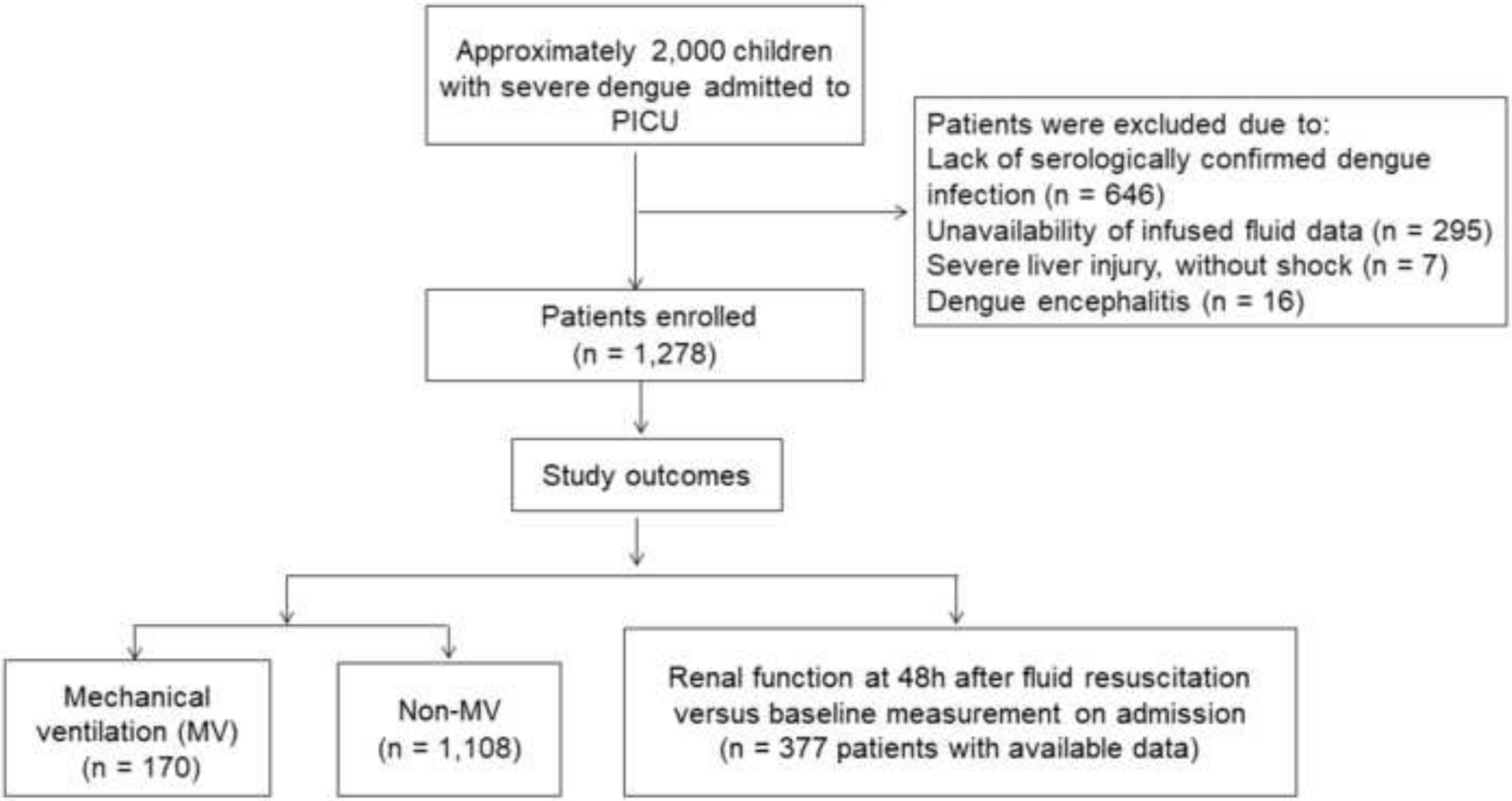

Click here to access/download;Figure;Fig2.tif

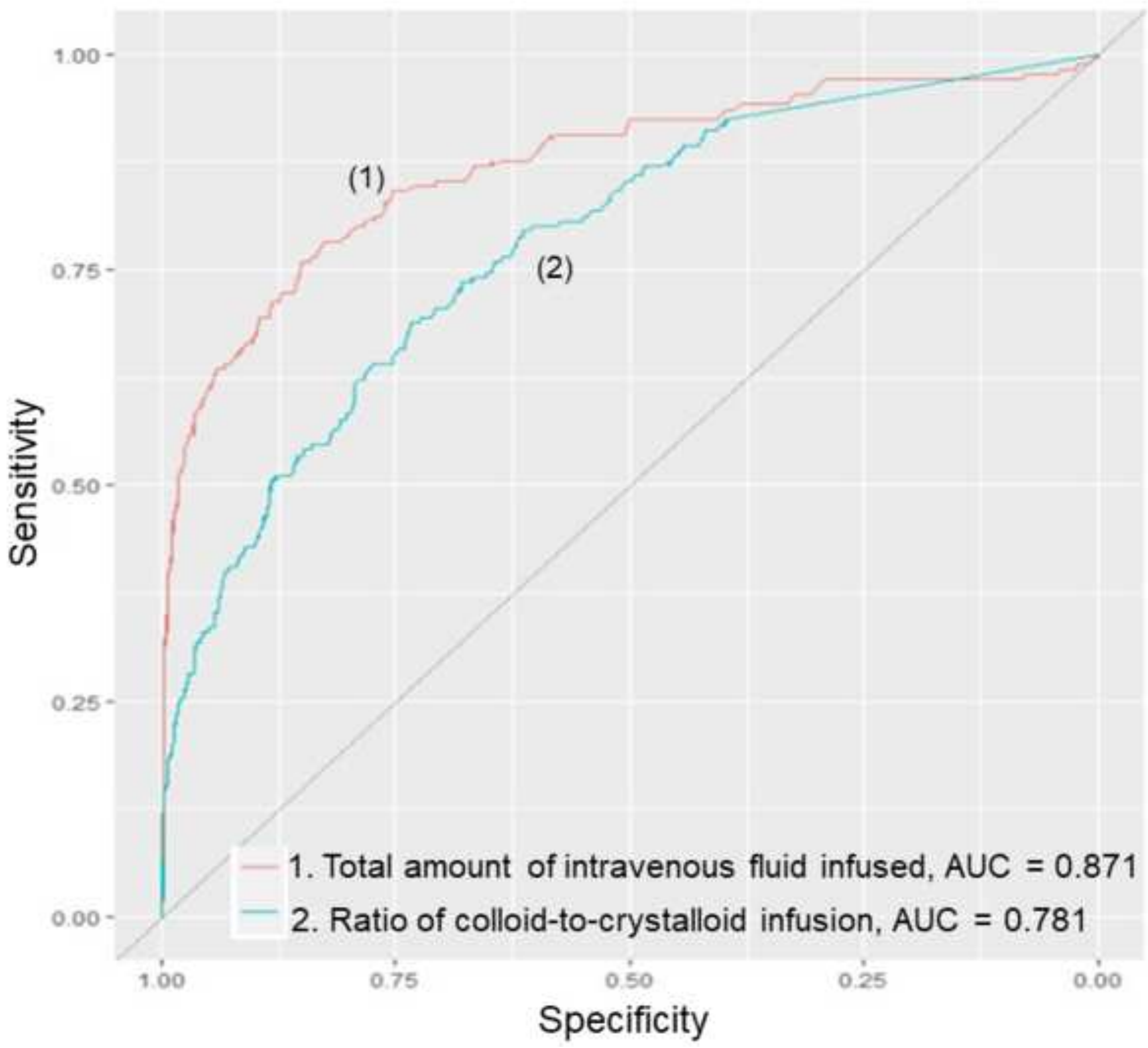

Fig3

Click here to access/download;Figure;Fig3.tif

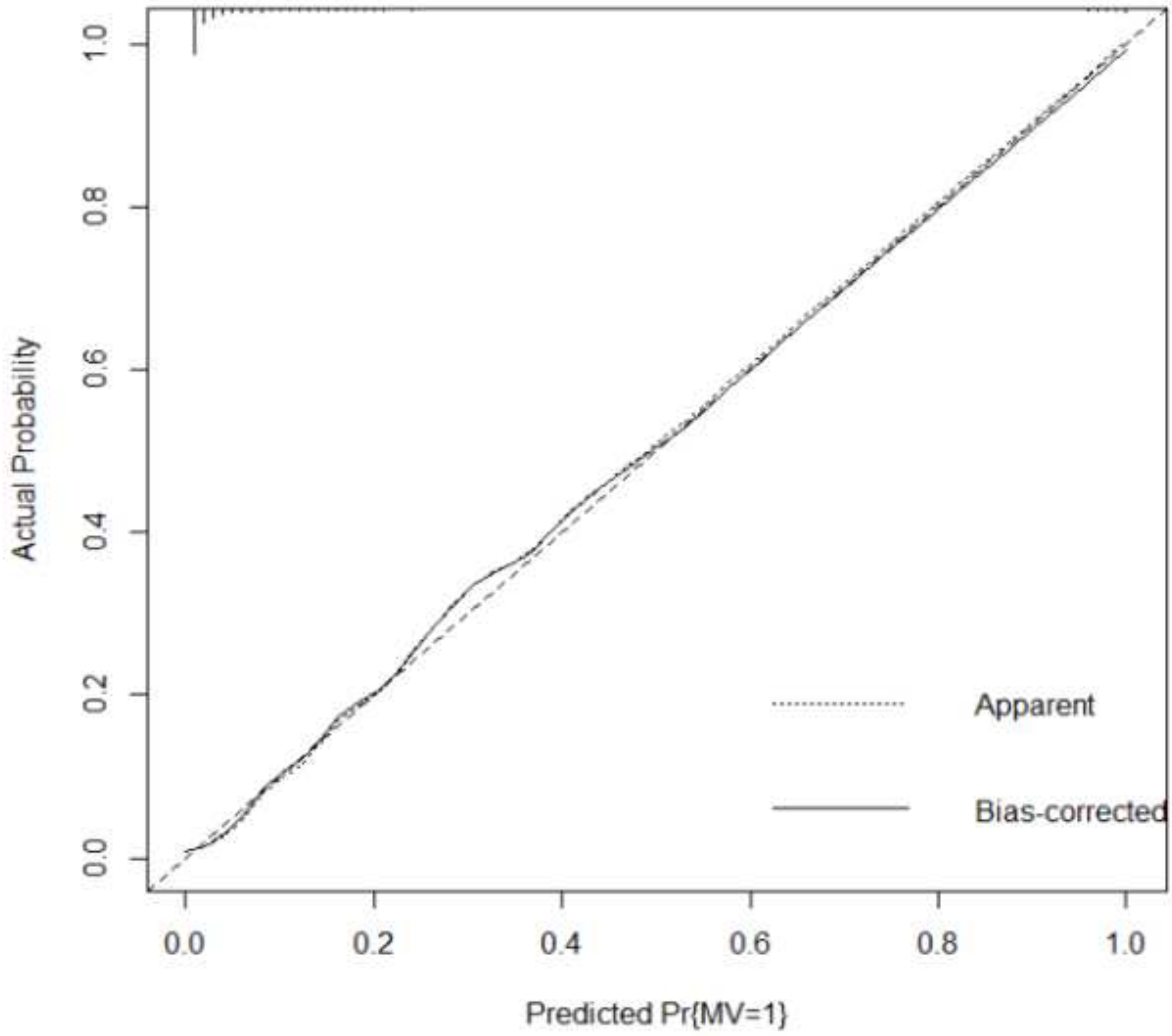

Fig4

Click here to access/download;Figure;Fig4.tif

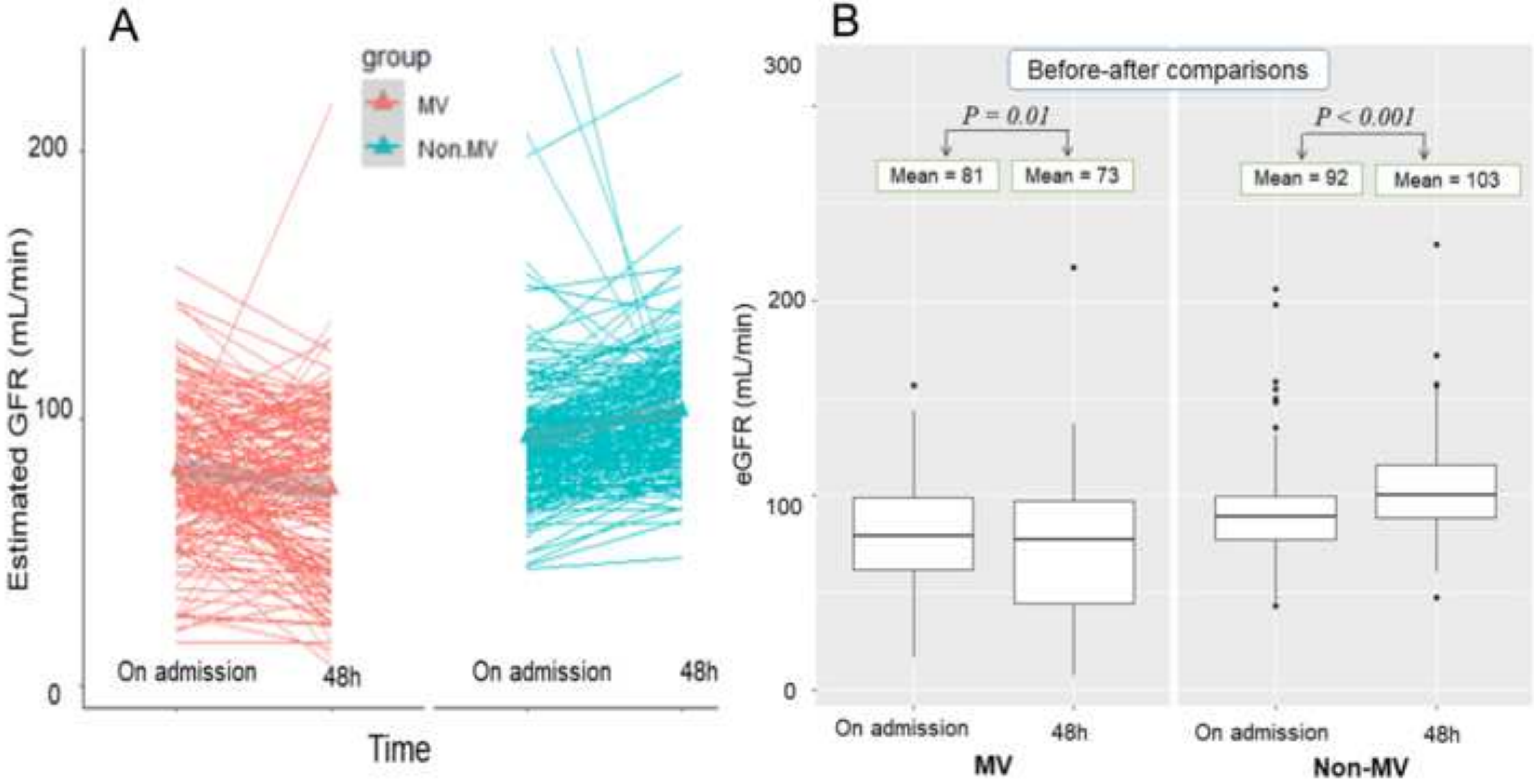

S1 Checklist

Click here to access/download
**Supporting Information**
S1 Checklist.doc

S1 File

Click here to access/download
**Supporting Information**
S1 File.docx

Click here to access/download
**Supporting Information - Compressed/ZIP File Archive**
S1 Appendix.rar
