# Supplementary material for: Associations of resuscitation fluid load, colloid-to-crystalloid infusion ratio and clinical outcomes in children with dengue shock syndrome
Source: PLoS Negl Trop Dis. 2025 Jan 10;19(1):e0012786. doi: 10.1371/journal.pntd.0012786 (PMC11756768; doi:10.1371/journal.pntd.0012786)
Supplement: Attachment — Submitted filename: Response Letter To Reviewers.docx [file pntd.0012786.s005.docx]

**Response letter to reviewers**

Dear Editors-in-Chief of the PLOS Neglected Tropical Diseases,

We are very thankful for your insightful comments for our submitted manuscript.

**MANUSCRIPT # PNTD-D-24-01370**, titled "**Associations of resuscitation fluid load, colloid-to-crystalloid infusion ratio and clinical outcomes in children with dengue shock syndrome**”

These are our responses to the reviewers’ comments from point to point.

**Reviewer's comments:**

**Key Review Criteria Required for Acceptance?**
As you describe the new analyses required for acceptance, please consider the following:

**Methods**
-Are the objectives of the study clearly articulated with a clear testable hypothesis stated?
-Is the study design appropriate to address the stated objectives?
-Is the population clearly described and appropriate for the hypothesis being tested?
-Is the sample size sufficient to ensure adequate power to address the hypothesis being tested?
-Were correct statistical analysis used to support conclusions?
-Are there concerns about ethical or regulatory requirements being met?

**Reviewer #1**: Yes

**Reviewer #2:** The objectives are clearly stated, study design is adequate, study population is well described, and statistical analyses seem appropriate.

Reviewer #3: (No Response)

**Reviewer #5**: (No Response)

**Reviewer #4**: Objectives are stated clearly but hypothesis is not stated.
Appropriate study design, Population is clear and appropriate, Sample size is sufficient, Statistical analysis is adequate, No ethical concerns

**Response:** We are thankful for all comments. We will add the study hypothesis in the revision manuscript, as suggestion from Reviewer 4, as below.

“*We hypothesized that larger volume of resuscitation fluid and more colloid than crystalloid infused would lead to poor clinical outcomes, including mechanical ventilation requirement and acute kidney injury among hospitalized children with DSS*.”

**(Lines 107-110**, in the *introduction* section of the revision manuscript)

**Results**
-Does the analysis presented match the analysis plan?
-Are the results clearly and completely presented?
-Are the figures (Tables, Images) of sufficient quality for clarity?

**Reviewer #1**: yes
**Reviewer #2**: Results are well presented, figures and tables are appropriate.
**Reviewer #3**: (No Response)

**Reviewer #5**: (No Response)

**Reviewer #4: Strengths:**
***Detailed Data***: The table captures a wide range of clinical and laboratory data, providing a comprehensive snapshot of the study population.
***Clear Metrics***: The use of median and interquartile ranges (IQR) for continuous variables ensures a clear understanding of data distribution.
Relevant Variables: Including key variables such as age, sex, DSS severity, and various clinical measures gives a thorough overview of the patients’ condition upon PICU admission.

**Areas for Improvement:**
*Elaborate on Significance*: It would be useful to include a brief explanation of why certain variables (like the systolic and diastolic shock indices or the high cumulative IV fluids) are significant and how they relate to patient outcomes.
*Comparative Context*: Providing comparative data or referencing other studies could help put these findings into context. How do these figures compare with similar studies or expected norms?
*Visual Aids*: Including charts or graphs alongside the table could help visualize key trends and differences, making the data more digestible. **Suggestions:**
Consider discussing the implications of these baseline characteristics for the prognosis or treatment strategies of DSS patients.
Discuss any potential biases introduced by the selection of participants and how these were managed or accounted for in the analysis.

**Response:** We will answer all questions from reviewers from point to point.

**### Reviewer 4:**

We are thankful for your comments. We will make appropriate amendments as your suggestion.

*Elaborate on Significance*: It would be useful to include a brief explanation of why certain variables (like the systolic and diastolic shock indices or the high cumulative IV fluids) are significant and how they relate to patient outcomes.

**Response:** Thank for these comments. We have included a brief explanation for such variables as the systolic and diastolic shock indices and the high cumulative IV fluids are significant, regarding their association with patients’ outcomes, as described below:

“*Systolic and diastolic shock indices were used to assess the dengue severity and the need for vasoactive inotrope support, as indicated higher values associated with more severity and vasopressor requirement*.”

(**Lines 155-157** in the revised manuscript)

-The high cumulative IV fluids and patient’s clinical outcomes

We have placed more emphasis on the importance of cumulative IV fluid and poor clinical outcomes of dengue mortality.

(**Lines 85-101** in the ***introduction*** in the revised manuscript)

*Visual Aids*: Including charts or graphs alongside the table could help visualize key trends and differences, making the data more digestible.

**Response:** We believe the tables and charts that we presented are sensible in scientific aspects. Currently, we believe we present sufficient charts to describe our main study objective. Therefore, we prefer not adding any further chart, because too many charts which may potentially distract the readers from our main study aims. We thank for your suggestion.

Consider discussing the implications of these baseline characteristics for the prognosis or treatment strategies of DSS patients.

**Response:** We are grateful for your insightful comments. In this study, we focused on the main study objectives of examining the association between the total volume of resuscitation fluid, colloid-to-crystalloid infusion ratio, and the outcomes of dengue patients (mechanical ventilation and acute kidney injury). Therefore, your suggestion is beyond our pre-specified study objectives. We think it may be appropriate in a future study with a greater focus on the prognosis and treatment strategies for patients with DSS. However, this is a very good point, and we consider your suggested points in a future study.

Discuss any potential biases introduced by the selection of participants and how these were managed or accounted for in the analysis.

**Response:** We thank the reviewers for their comment. We answered these queries comprehensively.

There were several sources of selection bias in this study.

- First, the main bias inherent to the retrospective study design was missing resuscitation fluid data from referred patients from provincial hospitals. This could potentially affect the primary outcome of mechanical ventilation) in both directions, toward or away from the null. To overcome this issue, only the participants with complete data were included in the final analysis.
- The second missing information source was serum creatinine level and patient height for eGFR estimation. This might potentially affect the analysis of secondary outcome (changes in kidney function). This could change the outcome in both directions, toward or away from the null. To address this issue, we only included complete cases in the statistical analysis.
- Another source of selection bias was the difference in disease severity, considering the patients referred from provincial hospitals (who had already received a large volume of resuscitation fluids) and those who were directly admitted to our hospital. The referred patient group commonly presented with greater disease severity. However, this is a real-life situation, and most importantly, enrolling both patient groups will increase the generalizability of the study.
- Finally, we strictly adhered to the inclusion and exclusion criteria to minimize selection bias.

We have presented these points in the statistical analysis and discussion section (final paragraph) of the manuscript.

**Conclusions**
-Are the conclusions supported by the data presented?
-Are the limitations of analysis clearly described?
-Do the authors discuss how these data can be helpful to advance our understanding of the topic under study?
-Is public health relevance addressed?

**Reviewer #1**: yes

**Reviewer #2:**

In the discussion section, the authors make several claims that cannot be directly inferred from the results. It is crucial to review certain sentences to align the conclusions with what was actually studied. While the formulated hypotheses are entirely plausible, it should be explicitly stated that they are only hypotheses addressing a complex phenomenon involving simultaneous inflammatory and hemodynamic changes, all contributing to the primary outcome: the requirement for mechanical ventilation. I would recommend more caution when interpreting therapeutic findings from observational studies, due to the numerous confounders present.

**Response:** Thank you for your comments. Based on our study findings, we generated several hypotheses regarding the pathogenesis of severe dengue. Your points are appreciated, and we will make appropriate amendments in the revised manuscript.

Predictors of severity should not be mistaken for causative factors.
Early in the discussion, the authors state that "*Resuscitation using macromolecular solutions is associated with increased mortality and acute kidney injury (AKI), whereas improved outcomes have been reported in patients treated with hyperoncotic albumin* [21]" (**line 326**). However, since the use of albumin is mentioned only in Table 2 as one of the blood products and not discussed earlier in the paper, emphasizing this hypothesis as an explanation for the results is unexpected.

**Response:** We thank the reviewer for their comment. This statement was based on the meta-analysis by Wiedermann et al. [21]. We did not indicate that our study yielded this significant point; instead, our study findings were aligned with a meta-analysis report [21]. We have made the following appropriate amendments using the words in the revised manuscript:

“*A meta-analysis has shown that resuscitation using macromolecular solutions is associated with increased mortality and acute kidney injury (AKI); nevertheless, better clinical outcomes have been reported in patients treated with hyperoncotic albumin* [21]"

**(Lines 331-334** in the ***Discussion***, in the revised manuscript)

**References:**

**[21]** Wiedermann CJ, Dunzendorfer S, Gaioni LU, Zaraca F, Joannidis M. Hyperoncotic colloids and acute kidney injury: a meta-analysis of randomized trials. Crit Care. 2010;14(5):R191. doi: 10.1186/cc9308. Epub 2010 Oct 28. PMID: 21029460; PMCID: PMC3219298.

In discussing the results (**line 332**), the authors appropriately note that their study demonstrates *total cumulative fluid infusion within the first 24 hours of PICU admission is a strong predictor of mechanical ventilation*. They point out that administering macromolecular solutions to severe DSS patients with extensive plasma leakage, who do not respond to initial crystalloid resuscitation, is often inevitable. Later, they state that “*the high volume of colloid solution poses a significant risk of severe respiratory failure, necessitating mechanical ventilation*” (**line 336**). However, it is not possible to assert that the volume of colloid is a cause of respiratory failure, or whether it is merely a predictor MV. The high volume of colloid solution may simply indicate severe disease, characterized by a high inflammatory response and significant plasma leakage.

**Response:** We thank the reviewer for the insightful comment. From the multivariable logistic regression (Table 3), there were many risk factors for mechanical ventilation (MV), including  cumulative infused fluid from referral hospitals and 24h PICU admission, ratio of colloid-to-crystalloid infusion, critical bleeding, severe transaminitis, female sex, and hematocrit.

To the best of our knowledge, we can only state that there is a strong association between total cumulative fluid infusion within the first 24 h of PICU admission and MV outcome. In other words, a high volume of colloid solution poses a significant risk of severe respiratory failure, and mechanical ventilation is required for these critically ill patients. In addition, we can say that a high volume of intravenous fluid infusion, with much higher colloid solution, is an independent predictor of MV outcome (from univariable and multivariable analyses adjusted for other covariates).

We did not conclude a cause-effect relationship because Hill’s criteria must be met for this conclusion [***References* 1,2**].

We have carefully rechecked our written sentences, and we believe they are appropriate for statistical statements.

**References:**

1. Perrio M, Voss S, Shakir SA. Application of the bradford hill criteria to assess the causality of cisapride-induced arrhythmia: a model for assessing causal association in pharmacovigilance. Drug Saf. 2007;30(4):333-46. doi: 10.2165/00002018-200730040-00006. PMID: 17408310.
2. Nowinski CJ, Bureau SC, Buckland ME, Curtis MA, Daneshvar DH, Faull RLM, Grinberg LT, Hill-Yardin EL, Murray HC, Pearce AJ, Suter CM, White AJ, Finkel AM, Cantu RC. Applying the Bradford Hill Criteria for Causation to Repetitive Head Impacts and Chronic Traumatic Encephalopathy. Front Neurol. 2022 Jul 22;13:938163. doi: 10.3389/fneur.2022.938163. PMID: 35937061; PMCID: PMC9355594.

Similarly, the finding that "*rising hematocrit was a protective factor against the need for MV*" (**line 345**) is insufficient to support the statement that "This indicates that *restricting IV fluids is recommended, despite the relatively elevated hematocrit levels*.", as it is not possible to assert that the elevation in hematocrit is a consequence of fluid restriction or merely a sign of clinical recovery, with improved renal function and hemodynamics, leading to a decreased need for fluid infusion.

**Response:** We thanked for this comment. The reviewer is right. We reviewed a report by Nguyen TT et al. in 2023 [Reference 2] and finding from this study, then we aimed to generate a hypothesis that rising hematocrit might be a protective factor against MV. This hypothesis needs to be tested in a future study with an adequate sample adjusted for potential confounders and other predictors withdrawn from this study. We have amended the appropriate sentences in the revised manuscript, as below:

“*Based on our findings and published reports [2], we assumed that increasing hematocit might be a protective factor against the need for MV, and restricting IV fluids has been proposed for mechanically ventilated DSS patients. These assumptions should be investigated in future studies*.”

**(Lines 351-354** in the ***Discussion*** in the revised manuscript)

**Reference**

[2] Nguyen TT, Nguyen DT, Vo TT, Dang OT, Nguyen BT, Pham DT, Nguyen TT, Duong YN, Doan DH, Nguyen TH, Ho LT, Nguyen PH, Phan DN, Tran TV, Nguyen TK, Luong DC, Pham AT, Dinh TT, Do VC, Vo LT. Associations of obesity and dengue-associated mortality, acute liver failure and mechanical ventilation in children with dengue shock syndrome. Medicine (Baltimore). 2023 Nov 17;102(46):e36054. doi: 10.1097/MD.0000000000036054. PMID: 37986332; PMCID: PMC10659721.

The same applies to the recommendation in **line 347** that *colloidal solutions should be minimized to ensure adequate hemoperfusion*. This hypothesis could be tested in future studies, but it is not a direct conclusion of the current study, as it was not evaluated.

**Response:** We are thankful for your comment. It is right that this was the hypothesis regarding colloidal solutions should be minimized to ensure adequate hemoperfusion. Based on the findings of this study, we developed this idea, which should be tested in future studies.

**In line 362,** instead of saying, "*The colloid-to-crystalloid ratio serves as a significant indicator for guiding colloid fluid resuscitation and suggests the optimal time to substitute colloid solutions with other beneficial fluids, such as hyperoncotic albumin*," it would be more accurate to say that “*the* *colloid-to-crystalloid ratio serves as a significant indicator for guiding colloid fluid resuscitation, and that future trials should explore the optimal colloid-to-crystalloid ratio and the role of other fluids, such as hyperoncotic albumin*.”

**Response:** We are very thankful for your suggested point. Your interpretation is clear, and we will make amendments as per your suggestions in the revised manuscript.

“*The colloid-to-crystalloid ratio serves as a significant indicator for guiding colloid fluid resuscitation, and future trials should explore the optimal colloid-to-crystalloid ratio and the role of other fluids such as hyperoncotic albumin.*”

**(Lines 368-370** in the ***Discussion*** in the revised manuscript)

Similarly, it is not possible to conclude that *the need for more blood transfusions among patients requiring MV support is due to the excessive use of macromolecular solutions*, as suggested by the phrase on **line 370**. More likely, these needs arise from a common cause explained by the pathophysiology of profound DSS. Furthermore, as albumin is among these blood products, it complicates the evaluation of this finding.

**Response:** We are very thankful for your insightful comment. We agree with your comments. It is correct that there are numerous unknown confounders that might affect the association between increased blood transfusion and MV support, and it cannot be concluded that “our results also indicated that more blood product transfusions were associated with MV support, possibly correlating with the overuse of macromolecular solutions.”

We have carefully reviewed this point, and we agree with the reviewer’s comment. We have removed this information from the revised manuscript.

**Reviewer #3**: (No Response)

**Reviewer #5**: (No Response)

**Reviewer #4:**

**Strengths:**
Clarity: You’ve succinctly highlighted the key findings and their clinical relevance.
Practical Application: Emphasizing the need for optimizing fluid management protocols demonstrates the importance of your research.

**Areas for Improvement:**
*Specificity*: Consider providing more specific recommendations based on your findings. For instance, detailing how fluid management protocols should be adjusted.
*Future Research*: Briefly mentioning areas for future research could strengthen the conclusion. This shows awareness of the study's limitations and an understanding of the broader research landscape.

**Response:** We are thankful for your comment. We have made the following amendments as per the reviewer’s suggestion in the revised manuscript:

“*This study has shown the significance of optimizing fluid management protocols among hospitalized children with severe DSS in improving clinical outcomes, including MV and acute kidney injury. Our study also raised several important questions: (i) rising hematocrit as a protective factor against MV, (ii) the beneficial role of hyperoncotic albumin in patients with severe DSS, and (iii) the optimal volumes of colloid and albumin solutions given to patients with DSS. Further investigation in prospective cohorts is essential to elucidate this knowledge gap, which is required to optimize the protocol of fluid resuscitation among patients with severe DSS*.”

**(Lines 450-457** in the ***Discussion*** in the revised manuscript)

**Editorial and Data Presentation Modifications?**
Use  this section for editorial suggestions as well as relatively minor modifications of existing data that would enhance clarity. If the only modifications needed are minor and/or editorial, you may wish to recommend “Minor Revision” or “Accept”.
**Reviewer #1**: I have added an attachment to the article with suggested edits for the author
Some errors in the article as outlined. Overall, The study is important and adds to the literature available for treatment or lack there of on the topic of DSS.
Limited impact and generalizability of the study results
**Reviewer #2**: I would recommend Minor Revision.
The results are relevant and methods seem adequate.
Although results are not sufficient to propose new treatment guidelines, they indicate prognostic predictors and can support future clinical trials.
Reviewer #3: (No Response)
Reviewer #4: Minor Revision
Reviewer #5: (No Response)

**Response:** We thank all the reviewers’ comments. These are our answer to your questions.

**Reviewer #1**: I have added an attachment to the article with suggested edits for the author
Some errors in the article as outlined. Overall, The study is important and adds to the literature available for treatment or lack there of on the topic of DSS.
Limited impact and generalizability of the study results

**Response:** We are very thankful for your comments. We thanked reviewer-1 for his/her enthusiasm in sending us suggestions for reediting, and we have made corrections as suggested in the revised manuscript. Our study aimed to generate hypotheses regarding fluid resuscitation in children with dengue shock syndrome. Our study raises new points for improving intravenous fluid management of DSS, which significantly supports future clinical trials.

**Summary and General Comments**
Use  this section to provide overall comments, discuss strengths/weaknesses of the study, novelty, significance, general execution and scholarship. You may also include additional comments for the author, including concerns about dual publication, research ethics, or publication ethics. If requesting major revision, please articulate the new experiments that are needed.
**Reviewer #1:** The study is important and adds to the literature available for treatment or lack there of on the topic of DSS but the disease is endemic to Asian countries, mostly South East Asia which limits the impact and generalizability of the study results.
The data is repetitive and a lot of the same things are mentioned in various different places in the study.

**Response:** We thanked for your comments. We have made appropriate amendments in the revised manuscript.

**Reviewer #2:** The paper addresses an important issue, analyzing a large case series of children with DSS. It clearly outlines the methods and acknowledges its limitations. The results are well-presented. However, the discussion section requires some revision. The findings related to prognostic predictors of fluid resuscitation parameters are crucial for clinical management and can inform future clinical trials, though they cannot be directly translated into treatment recommendations.

**Response:** Thank you for your insightful comments. We have made appropriate amendments to the discussion section of the revised manuscript.

**Reviewer #3:** The topic addresses a critical and specific area of pediatric care for DSS patients, adding valuable insights to the body of literature on dengue-related complications. This study is among the first to examine the colloid-to-crystalloid ratio as a predictor of mechanical ventilation in DSS, representing a novel and important contribution. It provides clear statistics, such as the fatality rate among patients requiring MV (22.4%) and the predictive value of the colloid-to-crystalloid ratio for MV requirement. The manuscript discusses the clinical implications of fluid resuscitation in DSS, particularly highlighting the risks of using large volumes of colloid solutions and the potential for acute kidney injury (AKI) and mechanical ventilation—crucial information for clinical practice. However, several points require further attention:
 - Some sentences are overly complex and could be simplified for better readability. For instance:
The sentence "*Resuscitation using macromolecular solutions is associated with increased mortality and acute kidney injury (AKI), whereas improved outcomes have been reported in patients treated with hyper-oncotic albumin*" can be rewritten for clarity.

**Response:** Thank you for your comments. We have made the following amendment as per your suggestion in the revised manuscript:

“*A meta-analysis has shown that resuscitation using macromolecular solutions is associated with increased mortality and acute kidney injury (AKI); however, better clinical outcomes have been reported in patients treated with hyperoncotic albumin* [21]"

**(Lines 331-334** in the **Discussion**, in the revised manuscript)

**Reference**

[21] Wiedermann CJ, Dunzendorfer S, Gaioni LU, Zaraca F, Joannidis M. Hyperoncotic colloids and acute kidney injury: a meta-analysis of randomized trials. Crit Care. 2010;14: R191. doi: 10.1186/cc9308. Epub 2010 Oct 28. PMID: 21029460; PMCID: PMC3219298.

- Additionally, consider breaking long paragraphs into smaller ones, especially when transitioning between key ideas. For example, the paragraph starting with "*In this study, 13.3% of the patients with DSS developed severe respiratory failure*..." should be divided when discussing different risk factors for mortality and mechanical ventilation.

**Response:** We thanked the reviewer for this comment. We have made amendment as suggestion in the revision manuscript, as below.

*“In this study, 13.3% of the patients with DSS developed severe respiratory failure requiring MV within 48 h of PICU admission. The overall in-hospital mortality rate was 3.1%.”*

**(Lines 325-326** in the revised manuscript.)

2.      The manuscript would benefit from more detailed descriptions of the cohort, particularly in terms of demographics and baseline health conditions. This additional context would help readers assess whether the findings can be generalized to broader populations. This is especially important given the mention of the patient population being derived from both provincial hospitals and direct admissions, which suggests heterogeneity in disease severity.

**Response:** We appreciate the reviewer’s comment. We collected the best participants’ data. This is a gentle reminder that this is a secondary data analysis from a retrospective study (2013-2022). We did not have detailed information on the referred patients from the provincial hospitals because of the lack of electronic medical records during the study period. This was inherent in the retrospective study design.

3.      While the limitations section is included, it should be expanded. The retrospective design is noted, but other potential confounders—such as variations in treatment protocols between different hospitals—should be discussed more thoroughly. The manuscript would also benefit from addressing selection bias due to the referral of more severe cases to the PICU. Moreover, the differences between patients referred from provincial hospitals and those admitted directly should be explored in more depth to assess how this variability might influence the study's findings.

**Response:** We thank for your comments. These are our answers to your questions.

- In our hospital, a tertiary pediatric hospital in Vietnam, we used similar management from the WHO dengue guidelines in 2009 [**Reference 7**] for treating hospitalized children with dengue shock syndrome over the study period.
- There were several sources of selection bias in this study.
- First, the main bias inherent to the retrospective study design was missing resuscitation fluid data from referred patients from provincial hospitals. This could potentially affect the primary outcome of mechanical ventilation) in both directions, toward or away from the null. To overcome this issue, only the participants with complete data were included in the final analysis.
- The second missing information source was serum creatinine level and patient height for eGFR estimation. This might potentially affect the analysis of secondary outcome (changes in kidney function). This could change the outcome in both directions, toward or away from the null. To address this issue, we only included complete cases in the statistical analysis.
- Another source of selection bias was the difference in disease severity, considering the patients referred from provincial hospitals (who had already received a large volume of resuscitation fluids) and those who were directly admitted to our hospital. The referred patient group commonly presented with greater disease severity. However, this is a real-life situation, and most importantly, enrolling both patient groups will increase the generalizability of the study.
- Finally, we strictly adhered to the inclusion and exclusion criteria to minimize selection bias.

These biases are inherent to the retrospective study design. It is gentle reminders that this is a secondary data analysis from a retrospective study (2013-2022). We did not have detailed information for the referred patients from the provincial hospitals because of lacking electronic medical records during study period time. We have already tried our best to capture all available data, as we could perform the analysis.

We have presented these points in the Statistical Analysis and Discussion section, in the revised manuscript.

**Reference**

[7] WHO. Dengue: Guidelines for Diagnosis, Treatment, Prevention and Control. Geneva: World Health Organization, 2009. Available from: <https://iris.who.int/handle/10665/44188>

4.      Although the discussion references other studies, such as those by Wang et al. and Diptyanusa et al., a clearer comparison between this study's findings and previous research would strengthen the manuscript. How do your results regarding AKI and MV align or contrast with existing studies? Providing this context could enhance the study's contribution to the field.

**Response:** We thank the reviewer for their comment. These are our answers to your questions.

Regarding MV outcome, there is a significant lack of data on mechanically ventilated patients with dengue shock syndrome. Several prior studies have reported that cumulative intravenous fluid overload exceeding 10-15% is highly associated with respiratory failure and mortality in children with severe dengue cases [References 2-6]. However, an accurate volume of intravenous fluid is unknown. In this regard, this study fills this knowledge gap.

In term of AKI outcome, the prevalence of AKI is low in our study cohort. Among DSS patients with AKI,  most experienced mild acute kidney injury and recovered well. This is illustrated in **Figure 4 in the manuscript submitted.**

**Figure 4**

In addition, the **Table 4** provided further insights into the AKI outcome of our study participants:

From the fixed-effect analysis in **Table 4** below, our study revealed that profound DSS, critical bleeding, and severe transaminitis are important risk factors for AKI events, which is consistent with the report by  Diptyanusa et al. [12]. We have also shown that AKI incidence was more prevalent in DSS patients who experienced critical respiratory failure requiring MV than in non-MV patients, consistent with a report by Wang et al. [14]. However, Wang et al. reported a higher proportion of underlying diseases, including hypertension, nephrotoxic drug use, and hematuria, as independent risk factors for AKI. In contrast, these factors were not observed in our pediatric cohort (age < 18 years).

Providing this context could enhance the study's contribution to the field.

**Response**: We have added these points to the revised manuscript as per the reviewer’s suggestion.

“*Notably, our study had distinct characteristics of participants compared to previous studies conducted by Wang et al. and Diptyanusa et al. [12,14].  First, our study cohort included pediatric patients (aged < 18 years) with DSS compared to adult dengue cohorts in those studies [12,14]. Second, in the study by Wang et al., a higher proportion of underlying diseases, including hypertension, nephrotoxic drug use, and hematuria, were independent risk factors for AKI. However, these factors were not observed in our pediatric cohort. In addition, our study revealed that profound DSS, critical bleeding, and severe transaminitis are significant risk factors for AKI events, which is consistent with the report by  Diptyanusa et al. [12]. Lastly, compared to previous studies, our study had the strength of describing the dynamic variation of kidney function (eGFR); however, the data were only available for 373 (of the total 1,278 patients). Hence, this study limitation impeded the accurate calculation and reporting of AKI prevalence in our patient cohort. Remarkably, our research group recently reported that the cumulative amount of infused fluid is a significant predictor of in-hospital mortality in children with DSS [15]. In this study, we further identified that a large cumulative amount of intravenous fluid infusion (≥ 181 mL/kg), particularly with a high proportion of colloid solutions, was associated with adverse dengue-related complications including mechanical ventilation requirement and acute kidney injury*.”

**(Lines 409-425** in the ***Discussion*** in the revised manuscript.)

**References**

[2] Nguyen TT, Nguyen DT, Vo TT, Dang OT, Nguyen BT, Pham DT, et al. Associations of obesity and dengue-associated mortality, acute liver failure and mechanical ventilation in children with dengue shock syndrome. Medicine (Baltimore). 2023; 102: e36054. doi: 10.1097/MD.0000000000036054. PMID: 37986332; PMCID: PMC10659721.

[3] Preeprem N, Phumeetham S. Paediatric dengue shock syndrome and acute respiratory failure: a single-centre retrospective study. BMJ Paediatr Open. 2022; 6: e001578. doi: 10.1136/bmjpo-2022-001578. PMID: 36645744; PMCID: PMC9668030.

[4] Vo LT, Nguyen DT, Tran TN, Tran HH, Đoan TT, Pham TN, et al. Pediatric Profound Dengue Shock Syndrome and Use of Point-of-Care Ultrasound During Mechanical Ventilation to Guide Treatment: Single-Center Retrospective Study, 2013-2021. Pediatr Crit Care Med. 2024; 25: e177-e185. doi: 10.1097/PCC.0000000000003413. Epub 2023 Nov 15. PMID: 37966344; PMCID: PMC10986784.

[5] Thanh NT, Dat NT, Thinh TN, Phuong NTM, Thanh MTH, Bao NT, et al. Therapeutic plasma exchange and continuous renal replacement therapy in pediatric dengue-associated acute liver failure: A case series from Vietnam. Transfus Apher Sci. 2023; 62: 103617. doi: 10.1016/j.transci.2022.103617. Epub 2022 Nov 28. PMID: 36522271.

[6] Sachdev A, Pathak D, Gupta N, Simalti A, Gupta D, Gupta S, et al. Early Predictors of Mortality in Children with Severe Dengue Fever: A Prospective Study. Pediatr Infect Dis J. 2021; 40 (9): 797-801. doi: 10.1097/INF.0000000000003179. PMID: 34321449

[12] Diptyanusa A, Phumratanaprapin W, Phonrat B, Poovorawan K, Hanboonkunupakarn B, Sriboonvorakul N, et al. Characteristics and associated factors of acute kidney injury among adult dengue patients: A retrospective single-center study. PLoS One. 2019; 14: e0210360. doi: 10.1371/journal.pone.0210360. PMID: 30615667; PMCID: PMC6322747.

[14] Wang C, Hong W, Ou Z, Yang H, Zhao L, Zhang Z, et al. Prevalence, Characteristics, and Outcomes Associated with Acute Kidney Injury among Adult Patients with Severe Dengue in Mainland China. Am J Trop Med Hyg. 2023; 109: 404-412. doi: 10.4269/ajtmh.22-0803. PMID: 37364862; PMCID: PMC10397446.

5.      The colloid-to-crystalloid ratio as a predictor of MV is a key point, but the discussion would benefit from a more detailed analysis of why a ratio of ≥ 1.6 is significant. How does this threshold compare with other fluid resuscitation guidelines? A more explicit discussion of potential cutoff points and alternative resuscitation strategies is needed.

**Response:** Thank you for your question. This question is answered in the following section.

We performed fluid resuscitation in patients with dengue shock syndrome (DSS) according to the 2009 WHO dengue guidelines. Crystalloids are recommended as first-line treatment for patients with DSS. If the patient does not respond to the initial crystalloids and/or in cases of prolonged severe DSS, colloidal solutions will be used. However, colloids have many adverse effects, such as coagulation disorders, reduced organ hemoperfusion, and increased risk of acute kidney injury. In addition, colloids damage the glycocalyx layer of vascular endothelial cells, exacerbating vascular leakage, and reducing intravascular flow and tissue hemoperfusion. At this timepoint, we believe switching to another beneficial solution such as hyperoncotic albumin is necessary. Evidence-based medicine to use hyperoncotic albumin is presented in the following [**question 6 below**]. One forward question is what clinical indicator should assist in this switching time point. However, to date, there are no guidelines for this query. In this study, we found an association between the increased colloid-to-crystalloid infusion ratio and the risk of mechanical ventilation in patients with DSS (adjusted OR = 2.2, 95% CI, 1.55-3.1, P < 0.001) (**Table 3,** in the submitted manuscript). We also performed sensitivity analysis and found the best cut-off point for the colloid-to-crystalloid infusion ratio and increased risk of MV ≥ 1.6  (**Table 2,** in the submitted manuscript).

Therefore, we propose a cut-off point ratio of colloid-to-crystalloid infusion ≥ 1.6 as an important indicator for switching from macromolecular solutions to more beneficial blood products such as albumin.

6.      The clinical recommendations regarding the minimization of colloid use and the potential role of hyperoncotic albumin are important but could be more specific. For instance, in which clinical scenarios should albumin be preferred over other fluids? Are there any guidelines or algorithms that clinicians should follow based on the study’s findings? Providing these details would make the recommendations more actionable.

**Response:** Thank you for your insightful comment. These are our answers to your questions. Currently, there are no international guidelines for the use of albumin in patients with severe DSS, even in the 2009 WHO dengue guidelines [1]. To date, data on the use of hyperoncotic albumin are limited in the population with severe dengue [2]. Lalitha et al. conducted a retrospective study on 76 children with DSS and showed that 20% albumin rapidly decreased lactate levels and shortened the duration of mechanical ventilation and hospital stay compared to 5% albumin [2]. We also gathered discrete evidence-based data to support the use of albumin [3-5].  Albumin potentially protects and maintains the integrity of the endothelial glycocalyx layer, reducing the rate of plasma leakage into interstitial tissues and restoring microcirculatory hemoperfusion [3]. In particular, concentrated albumin helps to reabsorb fluid from the interstitial tissue, restore circulatory volume, and reduce fluid demand [4,5].

A further question-In this scenario that albumin is preferred over other fluids?

**Response:**

In our hospital, we have been practicing using hyperoncotic albumin for patients with severe prolonged DSS with unstable hemodynamic conditions since 2023. Our clinical experience showed that better outcomes in patients with severe DSS were associated with the use of hyperoncotic albumin. Further prospective studies are necessary to examine the efficacy of hyperoncotic albumin therapy.

We based these data on the use of hyperoncotic albumin in patients with severely prolonged DSS with unstable hemodynamics, as follows:

Low albumin levels have been reported to be prognostic factors for severe DSS, and low albumin levels indicate a high rate of plasma leakage [1,6]. Very low levels of blood albumin are also observed in children with DSS who have prolonged shock with multiple organ failure; these patients have a high mortality rate of nearly 70% despite intensive treatment [7,8]. Therefore, albumin supplementation not only increases intravascular volume, restores oncotic pressure, and reduces the rate of fluid loss into the interstitial tissues but also supports the restoration of the glycocalyx layer, which is known to be the main factor causing increased vascular permeability in patients [9-12].

**References**

[1] WHO. Dengue: Guidelines for Diagnosis, Treatment, Prevention and Control. Geneva: World Health Organization, 2009. Available from: <https://iris.who.int/handle/10665/44188>

[2] Lalitha, A.V. Impact of resuscitation with 20% versus 5% albumin in outcome of severe dengue: a prospective observational study. Pediatric Critical Care Medicine. 2018; 19: p 36. [DOI: 10.1097/01.pcc.0000537423.59159.10]

[3] Aldecoa C, Llau JV, Nuvials X, Artigas A. Role of albumin in the preservation of endothelial glycocalyx integrity and the microcirculation: a review. Ann Intensive Care. 2020; 10: 85. doi: 10.1186/s13613-020-00697-1. PMID: 32572647; PMCID: PMC7310051.

[4] Zdolsek M, Hahn RG, Zdolsek JH. Recruitment of extravascular fluid by hyperoncotic albumin. Acta Anaesthesiol Scand. 2018; 62: 1255-1260. doi: 10.1111/aas.13150. Epub 2018 May 29. PMID: 29845612.

[5] Haynes, G.R., Bassiri, K. Hyper-oncotic vs. Hypo-oncotic Albumin Solutions: a Systematic Review of Clinical Efficacy and Safety. SN Compr Clin Med. 2021; 3: 1137–47. [Available from: <https://doi.org/10.1007/s42399-021-00755-0>].

[6] Sangkaew S, Ming D, Boonyasiri A, Honeyford K, Kalayanarooj S, Yacoub S, Dorigatti I, Holmes A. Risk predictors of progression to severe disease during the febrile phase of dengue: a systematic review and meta-analysis. Lancet Infect Dis. 2021 Jul;21(7):1014-1026. doi: 10.1016/S1473-3099(20)30601-0. Epub 2021 Feb 25. PMID: 33640077; PMCID: PMC8240557.

[7] Vo, Luan Thanh MD; Do, Viet Chau MD; Trinh, Tung Huu MD; Vu, Thien PhD; Nguyen, Thanh Tat MD. Combined Therapeutic Plasma Exchange and Continuous Renal Replacement Therapy in Children With Dengue-Associated Acute Liver Failure and Shock Syndrome: Single-Center Cohort From Vietnam*. Pediatric Critical Care Medicine 24(10):p 818-828, October 2023. | DOI: 10.1097/PCC.0000000000003304

[8] Kye Mon K, Nontprasert A, Kittitrakul C, Tangkijvanich P, Leowattana W, Poovorawan K. Incidence and Clinical Outcome of Acute Liver Failure Caused by Dengue in a Hospital for Tropical Diseases, Thailand. Am J Trop Med Hyg. 2016 Dec 7;95(6):1338-1344. doi: 10.4269/ajtmh.16-0374. Epub 2016 Oct 17. PMID: 27928082; PMCID: PMC5154447.

[9] Saravi B, Goebel U, Hassenzahl LO, Jung C, David S, Feldheiser A, Stopfkuchen-Evans M, Wollborn J. Capillary leak and endothelial permeability in critically ill patients: a current overview. Intensive Care Med Exp. 2023 Dec 20;11(1):96. doi: 10.1186/s40635-023-00582-8. PMID: 38117435; PMCID: PMC10733291.

[10] Durbin AP. Dengue vascular leak syndrome: insights into potentially new treatment modalities. J Clin Invest. 2019 Oct 1;129(10):4072-4073. doi: 10.1172/JCI131170. PMID: 31449055; PMCID: PMC6763244.

[11] Malavige GN, Ogg GS. Pathogenesis of vascular leak in dengue virus infection. Immunology. 2017 Jul;151(3):261-269. doi: 10.1111/imm.12748. Epub 2017 May 24. PMID: 28437586; PMCID: PMC5461104.

[12] Aldecoa C, Llau JV, Nuvials X, Artigas A. Role of albumin in the preservation of endothelial glycocalyx integrity and the microcirculation: a review. Ann Intensive Care. 2020 Jun 22;10(1):85. doi: 10.1186/s13613-020-00697-1. PMID: 32572647; PMCID: PMC7310051.

**Reviewer #4:** Overall Comments:
Comprehensive Coverage: Your paper does an excellent job of covering all necessary sections from the methodology to the results and conclusions. Each part is meticulously detailed, which is crucial for understanding the context and findings of your study.
Ethical Considerations: The ethical approval and adherence to the guidelines are well-documented. This strengthens the credibility of your research.
Data Analysis: Your statistical analysis is robust and well-justified. The use of various transformation and validation methods adds depth to your analysis, ensuring the reliability of your findings.
Clinical Relevance: The conclusions drawn are directly applicable to clinical practice, which is a significant strength. Your emphasis on optimizing fluid management protocols is particularly impactful.
Clear Presentation: Overall, your presentation of data, including tables and figures, is clear and easy to follow. However, integrating visual aids such as flowcharts or graphs to complement the data tables could enhance understanding.
**Suggestions for Improvement:**
Contextual Background: Providing a bit more context in the introduction about the significance of studying DSS in the population can help readers unfamiliar with the region or the condition.
Limitations: While you’ve mentioned missing data and the retrospective nature of the study, a more detailed discussion on the limitations and potential biases could be beneficial.
Future Research: Identifying areas for future research based on your findings can add value, highlighting how your study paves the way for further investigation.

Your research is well-structured and addresses important clinical questions. A few tweaks here and there, and you’re all set for a great impact!  Keep up the good work!

**Response:** Thank you for this comment. We have made all the appropriate amendments based on your suggestions in the revised manuscript.

**Reviewer #5:** COMMENTS
This retrospective study was conducted at a tertiary pediatric hospital in Vietnam from 2013 to 2022. It explores the associations and prognostic significance of resuscitation fluid load and, the colloid-to-crystalloid infusion ratio, in relation to clinical outcomes such as the need for mechanical ventilation and impaired renal function in children with dengue shock syndrome admitted to the PICU. The study provides valuable insights that can inform future randomized controlled trials in this vulnerable population.
**Specific comments:**
**Introduction:**
-       Discuss the ongoing debate regarding the ideal type and volume of fluids, as well as the optimal balance between colloid and crystalloid administration.

**Response:** We thank for your comments. We answer these questions as follows:

To date, fluid resuscitation for patients with dengue shock syndrome (DSS) has mainly been based on the 2009 WHO dengue guidelines [1]. Crystalloids are recommended as the first-line treatment for patients with DSS. If the patient does not respond to the initial crystalloids and/or in cases of severely prolonged DSS, colloidal solutions will be administered. Importantly, one primary mechanism of severe dengue infection is injury to the Endothelial Glycocalyx and Tight and Adherens Junctions [2,3], which induces progressive plasma leakage in patients with severe DSS. First, the Dengue viruses (with DENV NS1) directly disrupts the endothelial glycocalyx layer in human pulmonary microvascular endothelial cells, leading to hyperpermeability [4]. Second, a high volume of crystalloid fluid can potentially degrade the endothelial glycocalyx layer [5].

In addition, colloids will be used in patients who show a minimal response to crystalloids and progressive plasma leakage. However, colloids have many adverse effects, such as causing coagulation disorders, reduced hemoperfusion to organs, and increased risk of acute kidney injury. Colloids also damage the glycocalyx layer of vascular endothelial cells, exacerbating vascular leakage, and reducing intravascular flow and tissue hemoperfusion [6,7]. Wills et al. conducted a randomized controlled trial to compare three fluid solutions for resuscitation in dengue shock syndrome [8]. Wills et al. recommended initial resuscitation with Ringer's lactate for children with moderately severe DSS. Colloids such as dextran 70 and 6 percent hydroxyethyl starch are used similarly in children with severe shock [8].

A further question was raised: in profound DSS patients in whom both crystalloids and colloids are excessively administered, what are the appropriate solutions given to these critical DSS patients? Recently, albumin has been proposed; however, more evidence on its use is needed. To date, clinical data on the use of hyperoncotic albumin in patients with severe dengue are limited [9]. Lalitha et al. conducted a retrospective study on 76 children with DSS and showed that 20% albumin rapidly decreased lactate levels and shortened the duration of mechanical ventilation and hospital stay compared to 5% albumin [9]. Albumin potentially protects and maintains the integrity of the endothelial glycocalyx layer, reducing the rate of plasma leakage into interstitial tissues, and restoring microcirculatory hemoperfusion [10]. In particular, concentrated albumin helps to reabsorb fluid from the interstitial tissue, restore circulatory volume, and reduce fluid demand [11,12].

Therefore, to the best of our knowledge, both recommended crystalloids and colloids have both positive and negative effects on patients with DSS. The optimal balance of either crystalloids or colloids was based on a randomized controlled trial conducted by Wills et al. [8]. However, the accurate amounts of crystalloids and colloids that are effective in treating severe DSS are still unknown. Additionally, more evidence-based studies on albumin use are required. Further studies are needed to determine the ideal type and volume of fluids.

In alignment with the suggestion from the reviewer, we further added a short paragraph in the introduction in the revised manuscript (**Lines 88-103).**

**References**

[1] WHO. Dengue: Guidelines for Diagnosis, Treatment, Prevention and Control. Geneva: World Health Organization, 2009. Available from: <https://iris.who.int/handle/10665/44188>

[2]	Tejo AM, Hamasaki DT, Menezes LM, Ho YL. Severe dengue in the intensive care unit. J Intensive Med. 2023 Sep 28;4(1):16-33. doi: 10.1016/j.jointm.2023.07.007. PMID: 38263966; PMCID: PMC10800775.

[3] Suwarto S, Sasmono RT, Sinto R, Ibrahim E, Suryamin M. Association of Endothelial Glycocalyx and Tight and Adherens Junctions With Severity of Plasma Leakage in Dengue Infection. J Infect Dis. 2017 Mar 15;215(6):992-999. doi: 10.1093/infdis/jix041. PMID: 28453844; PMCID: PMC5407050.

[4] Puerta-Guardo H, Glasner DR, Harris E. Dengue Virus NS1 Disrupts the Endothelial Glycocalyx, Leading to Hyperpermeability. PLoS Pathog. 2016 Jul 14;12(7):e1005738. doi: 10.1371/journal.ppat.1005738. PMID: 27416066; PMCID: PMC4944995.

[5] Guidet B, Ait-Oufella H. Fluid resuscitation should respect the endothelial glycocalyx layer. Crit Care. 2014 Dec 23;18(6):707. doi: 10.1186/s13054-014-0707-6. PMID: 25629597; PMCID: PMC4331386.

[6] Dickenmann M, Oettl T, Mihatsch MJ. Osmotic nephrosis: acute kidney injury with accumulation of proximal tubular lysosomes due to administration of exogenous solutes. Am J Kidney Dis. 2008; 51: 491-503. doi: 10.1053/j.ajkd.2007.10.044. PMID: 18295066.

[7] Schortgen F, Girou E, Deye N, Brochard L; CRYCO Study Group. The risk associated with hyperoncotic colloids in patients with shock. Intensive Care Med. 2008; 34: 2157-68. doi: 10.1007/s00134-008-1225-2. Epub 2008 Aug 7. PMID: 18685828.

[8] Wills BA, Nguyen MD, Ha TL, Dong TH, Tran TN, Le TT, et al. Comparison of three fluid solutions for resuscitation in dengue shock syndrome. N Engl J Med. 2005; 353: 877-89. doi: 10.1056/NEJMoa044057. PMID: 16135832.

[9] Lalitha, A.V. Impact of resuscitation with 20% versus 5% albumin in outcome of severe dengue: a prospective observational study. Pediatric Critical Care Medicine. 2018; 19: p 36. [DOI: 10.1097/01.pcc.0000537423.59159.10]

[10] Aldecoa C, Llau JV, Nuvials X, Artigas A. Role of albumin in the preservation of endothelial glycocalyx integrity and the microcirculation: a review. Ann Intensive Care. 2020; 10: 85. doi: 10.1186/s13613-020-00697-1. PMID: 32572647; PMCID: PMC7310051.

[11] Zdolsek M, Hahn RG, Zdolsek JH. Recruitment of extravascular fluid by hyperoncotic albumin. Acta Anaesthesiol Scand. 2018; 62: 1255-1260. doi: 10.1111/aas.13150. Epub 2018 May 29. PMID: 29845612.

[12] Haynes, G.R., Bassiri, K. Hyper-oncotic vs. Hypo-oncotic Albumin Solutions: a Systematic Review of Clinical Efficacy and Safety. SN Compr Clin Med. 2021; 3: 1137–47. [Available from: <https://doi.org/10.1007/s42399-021-00755-0>].

-Conclude the introduction with a compelling statement highlighting the significance of the study. Emphasize why this research is essential and how it can help overcome the challenges of managing DSS in the PICU.

**Response:** We are thankful for this comment. As your suggestion, we have made the amendments in the last paragraph of introduction to conclude with a compelling statement highlighting the significance of the study in the revision paragraph, as below.

“*Previous studies have shown that severe respiratory failure requiring MV is a strong independent predictor of death in hospitalized children with severe dengue [2,3,6]. We have recently reported that cumulative infused fluid from referral hospitals and 24 hours of PICU admission was a significant risk factor for MV support [2]. Therefore, we hypothesized that a larger volume of resuscitation fluid and more colloids than infused crystalloids would lead to poor clinical outcomes, including mechanical ventilation requirement and acute kidney injury among hospitalized children with DSS.* *This study aimed to investigate the associations between resuscitation fluid load, the colloid-to-crystalloid infusion ratio, and clinical outcomes in children with DSS admitted to the PICU. A comprehensive understanding of fluid management strategies, particularly in patients with DSS requiring MV, is essential to improve survival outcomes. We aimed to establish an optimized fluid management protocol to minimize the need for MV and reduce the risk of kidney injury in pediatric DSS patients*.”

**(Lines 104-115** in the ***introduction*** in the revised manuscript)

**References**

[2] Nguyen TT, Nguyen DT, Vo TT, Dang OT, Nguyen BT, Pham DT, et al. Associations of obesity and dengue-associated mortality, acute liver failure and mechanical ventilation in children with dengue shock syndrome. Medicine (Baltimore). 2023; 102: e36054. doi: 10.1097/MD.0000000000036054. PMID: 37986332; PMCID: PMC10659721.

[3] Preeprem N, Phumeetham S. Paediatric dengue shock syndrome and acute respiratory failure: a single-centre retrospective study. BMJ Paediatr Open. 2022 ; 6(1): e001578. doi: 10.1136/bmjpo-2022-001578. PMID: 36645744; PMCID: PMC9668030

[6] Sachdev A, Pathak D, Gupta N, Simalti A, Gupta D, Gupta S, et al. Early Predictors of Mortality in Children with Severe Dengue Fever: A Prospective Study. Pediatr Infect Dis J. 2021; 40 (9): 797-801. doi: 10.1097/INF.0000000000003179. PMID: 34321449

**Methods and results:**
-       Since this is retrospective data, please mention the data source (electronic or paper-based), its reliability, and how the data was retrieved.

**Response:** We used paper-based data collection in CRF and then inputted it in an Excel file for further analysis with R. We have added your suggestions in the revised manuscript.

-       Excluding participants with missing data can introduce selection bias, as those included in the analysis may differ systematically from those excluded.

**Response:** We thanked for this comment. In this study, as shown in **Figure. 1.** Of the 2,000 children admitted to the PICU during the study period, we excluded 295 (approximately 15%) who did not have adequate infusion data, and the remaining patients were excluded due to lack of serological confirmation for dengue infection. We agree with the reviewer that, in theory, this may induce selection bias, but we do believe that this had a small effect because of a relatively low proportion of missing data (15%). Most importantly, we performed a sensitivity analysis that required a complete case dataset. Additionally, we also performed bootstrap resampling (n=500) and internal validation, and these statistical analytic methods have been shown to manage the proportion of missing data in this study and improve study power.

**Figure 1**

-       When describing the models, provide a brief explanation for the inclusion of specific variables. For instance, clarify why variables like severe bleeding, severe transaminitis, hematocrit, and platelet counts were chosen. This helps readers understand the rationale behind your model choices.

**Response:** We thanked for this comment. These are our answers.

We did describe a predefined set of covariates chosen in the model from clinical knowledge, expertise in this field, and a medical literature review in the manuscript.

***Study outcomes and candidate variables***

“*The main study outcomes were mechanical ventilation requirement during the PICU stay and changes in renal function within 48 h after PICU admission. A predefined set of covariates was predetermined, including age, sex, DSS severity, severe bleeding, severe transaminitis, platelet counts, hematocrit levels, cumulative infused fluid volume within the first 24 h of PICU admission, and percentages of colloid and crystalloid fluids. These covariates were preselected based on our clinical knowledge, disease pathogenesis and medical literature* [2,4,15].”

**(Lines 141-147** in the **Methods** in the revised manuscript)

- The severity of DSS, as well as the group referred from other hospitals, could be potential confounders that might influence the need for mechanical ventilation or the risk of kidney injury.

**Response:** We thanked for the reviewer’s comment. We considered that DSS severity might be a potential confounding factor. However, we based our recent publications [**References 1,2**] on excluding the DSS severity as a confounder for MV. In those studies, we performed multiple logistic regression adjusted for DSS severity and found that it was neither a risk factor nor a confounder of MV outcome.

Regarding the severity of DSS for the risk of kidney injury, we performed a linear mixed effect model **(Table 4** in the submitted manuscript) adjusted for profound DSS (indicating DSS severity) in the modelling. Regression for a factor was reported as a method to check whether a variable was a confounder. Fixed effect analysis showed that profound DSS (severe prolonged DSS) was neither a significant predictor of kidney injury nor a confounder.

Notably, based on the disease pathogenesis, dengue is an acute infection, and referred patients were most frequently treated for 24 h in provincial hospitals and then rapidly transferred to our tertiary hospital in cases of severe dengue. In this short period, the effect of  DSS severity in the referred group was not clinically significant.

**References**

[1] Nguyen Tat T, Vo Hoang-Thien N, Nguyen Tat D, Nguyen PH, Ho LT, Doan DH, Phan DT, Duong YN, Nguyen TH, Nguyen TK, Dinh HT, Dinh TT, Pham AT, Do Chau V, Trinh TH, Vo Thanh L. Prognostic values of serum lactate-to-bicarbonate ratio and lactate for predicting 28-day in-hospital mortality in children with dengue shock syndrome. Medicine (Baltimore). 2024 Apr 26;103(17):e38000. doi: 10.1097/MD.0000000000038000. PMID: 38669370; PMCID: PMC11049702.

[2] Nguyen TT, Nguyen DT, Vo TT, Dang OT, Nguyen BT, Pham DT, Nguyen TT, Duong YN, Doan DH, Nguyen TH, Ho LT, Nguyen PH, Phan DN, Tran TV, Nguyen TK, Luong DC, Pham AT, Dinh TT, Do VC, Vo LT. Associations of obesity and dengue-associated mortality, acute liver failure and mechanical ventilation in children with dengue shock syndrome. Medicine (Baltimore). 2023 Nov 17;102(46):e36054. doi: 10.1097/MD.0000000000036054. PMID: 37986332; PMCID: PMC10659721.

-  Please clarify how the authors determined the sample size required to detect statistically significant effects between resuscitation fluid load, the colloid-to-crystalloid infusion ratio, and clinical outcomes in children with DSS. Alternatively, conduct a power analysis to assess whether the dataset is sufficient to test the hypotheses adequately.

**Response:** We thank for the reviewer’s comment. We will answer your questions.

In this study, the primary outcome was mechanical ventilation (MV), and the secondary outcome was acute kidney injury (AKI). BMJ 2020, doi: <https://doi.org/10.1136/bmj.m441> ) [1]. As a rule of thumb, the sample size was required to ensure at least 10 events for each predictor parameter. Our study had eight predefined covariates; hence, 8 × 10 = 80 events (patients with MV outcomes) were required. According to our recent publication [2], mechanical ventilation in children with DSS was 13% [2]. Therefore, a sample size of 615 patients was needed [(80 × 100) / 13]. In our study, there were 170 MV outcome events (13.3%), and total participants was 1,278 included in the analysis. This highlights the adequate study power of the predictive MV model developed.

Second, for the secondary outcome of AKI, we mainly aimed to explore the variation in eGFR by using linear effect modelling. The sample size calculation was sophisticated, and this was an exploratory study.

Finally, to the best of our knowledge, up-to-date advanced statistics with resampling methods (bootstrap and oversampling using the SMOTE method [3]) can aid in resampling datasets. This significantly reduces the pressure from the sample size requirement of conventional statistics.

**Reference:**

# [1] Richard D Riley et al. Calculating the sample size required for developing a clinical prediction model. BMJ 2020. doi: <https://doi.org/10.1136/bmj.m441>

[2] Nguyen TT, Nguyen DT, Vo TT, Dang OT, Nguyen BT, Pham DT, Nguyen TT, Duong YN, Doan DH, Nguyen TH, Ho LT, Nguyen PH, Phan DN, Tran TV, Nguyen TK, Luong DC, Pham AT, Dinh TT, Do VC, Vo LT. Associations of obesity and dengue-associated mortality, acute liver failure and mechanical ventilation in children with dengue shock syndrome. Medicine (Baltimore). 2023 Nov 17;102(46):e36054. doi: 10.1097/MD.0000000000036054. PMID: 37986332; PMCID: PMC10659721.

[3] Sowjanya AM, Mrudula O. Effective treatment of imbalanced datasets in health care using modified SMOTE coupled with stacked deep learning algorithms. Appl Nanosci. 2023;13(3):1829-1840. doi: 10.1007/s13204-021-02063-4. Epub 2022 Feb 3. PMID: 35132368; PMCID: PMC8811587.

-       The authors mentioned that no significant interactions were found among the covariates, but no data was provided to substantiate this claim.

**Response:** We thanked for this comment. First, based on clinical knowledge, we did not observe a potential interaction based on predefined covariates. Second, we performed statistical analysis and found no interaction among the covariates, as stated in the submitted manuscript. Considering the negative findings that had no impact on our interpretation of the main study results, we preferred not to report detailed interaction analysis because we believe it may distract the readers from our main findings as we presented.

We show the R outputs for testing the interactions among covariates.

As presented in the R output below, there was no significant interaction between the total amount of fluid infused and  other covariates.

**Checking interactions-Total IV fluid infused (log-2 normalization) with other covariates:**

**Checking interactions-Severe bleeding with other covariates**

Our analysis showed no significant interactions between severe bleeding and other covariates, in combination with our clinical knowledge.

In Table 4, the authors reported that the random effects analysis revealed an intra-group correlation of 5%, but no supporting documentation was provided. The authors should include the full model as a supplementary table so that interested readers can examine the results in detail.

**Response:** Thank you for your comments. These data can be conveniently drawn from Table 4 using a simple calculation.

Extract from Table 4, mainly focusing on random effects analysis, as follows:

The random effect (intra-class / intra-mechanical ventilation correlation) can be calculated by this formula: = 39 / (39+721) = 39 / 760 = 5 % (as we presented and discussed in the manuscript).

This calculation is common with researchers who have a background of linear mixed-effect modelling. Thus, we do not intend to add a supplementary table as suggested.

*Additionally, the complete* ***Table 4*** *in the submitted manuscript is shown below:*

**Discussion**

**Response:** We are thankful for your comments. We will answer from point to point in the following:

1. Avoid referencing tables in the discussion section:

**Response:** Thank for this comment. We have made amendment as your suggestion in the revised manuscript.

2. The authors identified female gender as a significant predictor of mechanical ventilation in DSS patients, which requires further explanation.

**Response**: Several meta-analyses and systematic reviews have shown that female patients have a higher risk of developing severe dengue than comparable male patients [**References: 1,2**]. We used this result to input sex as a significant risk factor for the mechanical outcome. As expected, our clinical data analysis revealed that female patients with DSS had 1.8 times of higher risk of developing MV. This stems from published clinical studies; however, the exact pathogenesis remains unclear.

**References**

[1] Sangkaew S, Ming D, Boonyasiri A, Honeyford K, Kalayanarooj S, Yacoub S, Dorigatti I, Holmes A. Risk predictors of progression to severe disease during the febrile phase of dengue: a systematic review and meta-analysis. Lancet Infect Dis. 2021 Jul;21(7):1014-1026. doi: 10.1016/S1473-3099(20)30601-0. Epub 2021 Feb 25. PMID: 33640077; PMCID: PMC8240557.

[2] Paraná VC, Feitosa CA, da Silva GCS, Gois LL, Santos LA. Risk factors associated with severe dengue in Latin America: A systematic review and meta-analysis. Trop Med Int Health. 2024 Mar;29(3):173-191. doi: 10.1111/tmi.13968. Epub 2024 Jan 23. PMID: 38263345.

3. Discuss how these data can be helpful to advance our understanding of fluid management in DSS.

Discuss the public health relevance of the study results?

**Response:** We thank for your comments. We answer these questions as follows.

At our tertiary hospital, we performed fluid resuscitation for patients with dengue shock syndrome (DSS) according to the 2009 WHO guidelines. Crystalloids are recommended as the first-line treatment for patients with DSS. If the patient does not respond to the initial crystalloids, and/or in cases of prolonged severe DSS, we will use colloid solutions. However, colloids have many adverse effects, such as causing coagulation disorders, reduced hemoperfusion to organs, and increased risk of acute kidney injury. In addition, colloids damage the glycocalyx layer of vascular endothelial cells, exacerbating vascular leakage and reducing intravascular flow and tissue hemoperfusion. At this time point, we believe that switching to another beneficial solution, such as hyperoncotic albumin, is recommended.

Importantly, the current available studies and the 2009 WHO dengue guidelines have shown that a large volume of intravenous fluid administration, particularly high-dose colloid solutions in treating patients with DSS, can potentially lead to many adverse events (as stated above). However, specific figures for the total volume of intravenous fluid and the threshold that colloids can cause serious poor clinical outcomes are still unclear.

From the sensitivity analysis (**Table 2** in the submitted manuscript), our study clearly showed that the total volume of intravenous fluid administration for children with DSS ≥ 181 mL/kg significantly increases the risk of respiratory failure requiring mechanical ventilation. Importantly, the cutoff colloid-to-crystalloid infusion ratio of ≥ 1.6 increased the risk of severe respiratory complications requiring MV in patients with DSS.

Therefore, our study has significantly filled the existing knowledge gap regarding fluid resuscitation for children with DSS. Notably, the study population in our study included children with DSS, which has been widely documented as the most frequent complication in patients with severe dengue and as a contributing factor to a high fatality rate (up to 20%). Our study can aid in optimizing fluid resuscitation schemes in DSS patients to minimize poor dengue-related outcomes, particularly in WHO-reported regions with the highest dengue-related burdens, particularly in South America, Africa, and Southeast Asia.

4. Consider discussing future research directions, for example, the types of colloid and crystalloid fluids to be used for resuscitation and the optimal timing for administering each.

**Response:** Thank you for this comment. We have amended this suggestion in the revised manuscript.

**“***This study has shown the significance of optimizing fluid management protocols among hospitalized children with severe DSS in improving clinical outcomes, including MV and acute kidney injury. Our study also raised several important questions: (i) permissive elevated hematocrit level and clinical outcomes in patients with severe dengue, (ii) the beneficial role of hyperoncotic albumin in patients with severe DSS, and (iii) the optimal volumes of colloid and albumin solutions given to patients with severe DSS. Further investigation in prospective cohorts is essential to elucidate this knowledge gap, which is required to optimize the protocol of fluid resuscitation among patients with severe DSS*.”

**(Lines 450-457** in the ***Discussion*** in the revised manuscript)

**Journal editor’s comments:**

**1. Figure Files:**
While revising your submission, please upload your figure files to the Preflight Analysis and Conversion Engine (PACE) digital diagnostic tool, [https://pacev2.apexcovantage.com](https://pacev2.apexcovantage.com/). PACE helps ensure that figures meet PLOS requirements. To use PACE, you must first register as a user. Then, login and navigate to the UPLOAD tab, where you will find detailed instructions on how to use the tool. If you encounter any issues or have any questions when using PACE, please email us at [figures@plos.org](mailto:figures@plos.org).

**Response:** Yes, we are thankful for this information and we checked the figures as requirements. However, we have encountered resizing of the figures as the journal required. We expect to receive assistance from the editorial team for the format of the figures.

 **2.** **Data Requirements:**
Please note that, as a condition of publication, PLOS' data policy requires that you make available all data used to draw the conclusions outlined in your manuscript. Data must be deposited in an appropriate repository, included within the body of the manuscript, or uploaded as supporting information. This includes all numerical values that were used to generate graphs, histograms etc.. For an example see here: <http://www.plosbiology.org/article/info%3Adoi%2F10.1371%2Fjournal.pbio.1001908#s5>.
**Response:** Yes, we have strictly followed the journal requirements regarding this issue.

**3. Reproducibility:**
To enhance the reproducibility of your results, we recommend that you deposit your laboratory protocols in [protocols.io](http://protocols.io/), where a protocol can be assigned its own identifier (DOI) such that it can be cited independently in the future. Additionally, PLOS ONE offers an option to publish peer-reviewed clinical study protocols. Read more information on sharing protocols at <https://plos.org/protocols?utm_medium=editorial-email&utm_source=authorletters&utm_campaign=protocols>

**Response:** This is not applicable to our study.

We have answered all the questions by the five reviewers, and we have made amendments as suggested.

We are thankful for your comments that have made our study more comprehensive for the readers.

We look forward to your feedback and answer any further questions.

Many thanks,

Best regards,

Dr Thanh Nguyen Tat
